# Collagen type 1 promotes survival of human breast cancer cells by overexpressing Kv10.1 potassium and Orai1 calcium channels through DDR1-dependent pathway

**DOI:** 10.18632/oncotarget.19065

**Published:** 2017-07-08

**Authors:** Mehdi Badaoui, Cloé Mimsy-Julienne, Charles Saby, Laurence Van Gulick, Marta Peretti, Pierre Jeannesson, Hamid Morjani, Halima Ouadid-Ahidouch

**Affiliations:** ^1^ Laboratory of Cellular and Molecular Physiology, EA4667, University of Picardie Jules Verne, Amiens, France; ^2^ Extracellular Matrix and Cellular Dynamics, Faculty of Pharmacy, MEDyC Centre National de la Recherche Scientifique UMR7369, Reims University, Reims, France

**Keywords:** Kv10.1, Orai1, tumour microenvironment, survival, breast cancer

## Abstract

Collagen type 1 is among the tumor microenvironment (TM) factors, that regulates proliferation, survival, migration and invasion. Ion channels are key players in interactions between tumor cells and TM. Kv10.1 has been shown to play an essential role in breast cancer cell proliferation and migration by permitting Ca^2+^ influx notably via Orai1. Here, we show that human breast cancer (BC) cells growing, in culture media completely devoid of the serum and seeded on collagen 1 coating, exhibited less apoptotic rate and a decrease in Bax expression when compared to those grown on plastic. The survival conferred by collagen 1 was completely abolished by removing extracellular Ca^2+^ from the culture medium. In addition, Ca^2+^ entry was increased in collagen 1 condition along with increased Kv10.1 and Orai1 expressions. Moreover, collagen 1 was able to increase co-localization of Kv10.1 and Orai1 on the plasma membrane. Interestingly, silencing of Kv10.1 and Orai1 reduced survival and Ca^2+^influx without any additive effect. This calcium-dependent survival is accompanied by the activation of ERK1/2, and its pharmacological inhibition completely abolished the increase in Kv10.1 and Orai1 expressions, activities, and the cell survival induced by collagen 1. Moreover, both Kv10.1 and Orai1 knockdown reduced ERK1/2 activation but not Akt. Finally, DDR1 silencing but not β1-integrin reduced the collagen induced survival, ERK1/2 phosphorylation and the expression of Kv10.1 and Orai1. Together these data show that the Kv10.1/Orai1 complex is involved in BC cell survival and this is dependent on collagen 1/DDR1 pathway. Therefore, they represent a checkpoint of tumor progression induced by the tumor microenvironment.

## INTRODUCTION

The tumor microenvironment controls various aspects of the tumor cell behaviour such as proliferation, apoptosis, migration, and invasion [1]. It contains a large number of cellular matrix proteins that regulate many of the tumor cell functions through the interaction of a peptide sequence with its specific receptor and transduction of intracellular signalling [2]. Among these proteins collagen type 1 (collagen 1) is the most abundant in mammals playing an essential role in the maintaining of the tissue integrity [3]. However, several studies have demonstrated that this protein is able to interact with several cell surface receptors and regulates intracellular signalling in pathological conditions. Depending on tumor and cell culture models, collagen 1 is able to downregulate cell proliferation [4], induce apoptosis [5], or promote survival and protect cancer cells against chemotherapy [6, 7]. Among the collagen 1 receptors, integrins are the most studied [8], particularly α1β1 and α2β1 heterodimers, which recognize the sequence GFOGER [9]. Otherwise, the Discoidin-Domain Receptors (DDR1 and DDR2) have been also explored in the past two decades [10]. DDR1 and DDR2 receptors differ from integrins in that they are the only receptors of collagen 1 harboring tyrosine kinase activity and recognizing the GVMGFO sequence of collagen 1 [11]. Unlike conventional tyrosine kinase receptors with a rapid and transient activation, DDR1 and DDR2 receptors exhibit a relative late and prolonged activation [12]. The role of these receptors in cell proliferation [4], apoptosis [13], invasion [14] and cell survival [15] has been reported in human fibrosarcoma, breast, lung, and colorectal cancers respectively. Breast cancer is characterized by a dense reactive stroma associated with extensive collagen 1 deposit [16]. Alterations in structural organization of collagen 1 occur during the first state of breast cancer (BC) development and promote local invasion [17]. Moreover, a relationship between collagen 1 density and the capacity of the development of BC has been reported [18].

During the past 20 years, several studies have demonstrated the association between the deregulation of the expression and/or activity of ion channels and the development of different types of cancer, and considerable progress has been made in understanding the role of ion channels in cancer [19, 20, 21]. Among these channels, Kv10.1, a member of the EAG family, has been mostly studied in cancer. Indeed, Kv10.1 has been reported to have oncogenic properties [22]. Interestingly, its expression in normal tissues is mainly restricted to the brain [23]. However, it is overexpressed in primary solid tumors including breast cancer [23]. In MCF-7 BC cells, Kv10.1 regulates the cell cycle progression, where it allows the entry of the non-invasive BC cells in G1 phase by hyperpolarizing the membrane potential and increasing the cytosolic Ca^2+^ concentration [24, 25]. Moreover, Kv10.1 regulates also the serum-induced migration in MDA-MB-231 mammary cancer cells via a Ca^2+^ entry through Orai1 channel [26]. Emerging studies suggest Kv10.1 as a key player in interactions between tumor cells and TM. Indeed, Toral and colleagues have demonstrated that CHO cells over-expressing Kv10.1 displayed a reorganization of cytoskeleton and lamellipodia and stress fiber formation when the cells were grown on collagen, while wide type CHO did not show this process. This seems to be specific to collagen since the observed processes did not occur in Kv10.1-CHO cells grown on poly-lysine [27]. While many studies including ours, have focused on the role of Kv10.1 in cancerous cells, very little data are available on the specific function of Kv10.1 channel in tumor regulation by cell microenvironment. In this study, we investigated whether Kv10.1 channel is involved in BC cell survival induced by collagen 1, and the molecular mechanisms involved. We show, for the first time, that collagen 1 is able to induce cell survival in fetal calf serum-starved BC cells through ERK1/2 phosphorylation and the overexpression of both Kv10.1 and Orai1 ion channels. Moreover, collagen 1 was able to increase Kv10.1 and Orai1 co-localization in the plasma membrane. We suggested that this co-localization may allow Orai1 channel to increase the basal Ca^2+^ influx mediated by Kv10.1, triggering ERK1/2 activation and cell survival. Interestingly, the DDR1 (a potential target of collagen 1) knockdown induced a decrease in ERK1/2 phosphorylation and expression of both Kv10.1 and Orai1, and consequently an increase in apoptosis. Collectively, our findings highlighted a matrix-dependent function for Kv10.1 in cell survival mediated by collagen 1.

## RESULTS

### Collagen 1 promotes BC cell survival

**Figure 1:**
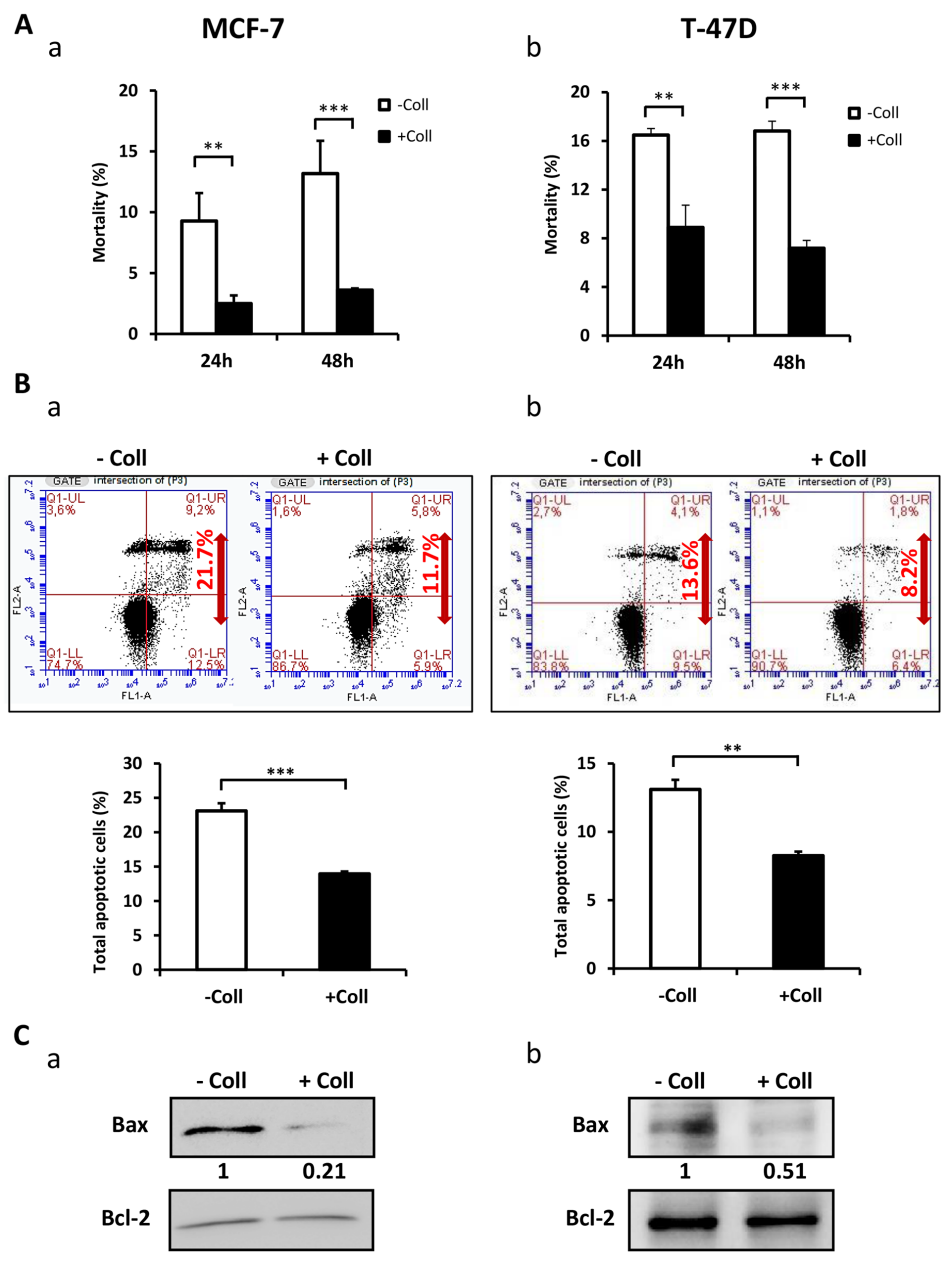
Collagen 1 induced MCF-7 and T-47D cells survival under serum starvation **(A)** Effect of collagen 1 on BC cell survival. The cell mortality was measured 24 h and 48 h post-starvation by Trypan Blue in MCF-7 (a) and T-47D (b) cells. **(B)** Measurement of rate of apoptosis in plastic and collagen 1 conditions 48 h post-starvation by Annexin V staining in MCF-7 (a) and T-47D (b) cells. **(C)** Representative western blot showing Bax and Bcl-2 expression in MCF-7 (a) and T-47D (b) cells 48 h post-starvation. Results were normalised as a percentage of the control condition (without collagen 1). Values are reported as mean ± SEM of triplicate experiments. ^**^p < 0.01, ^***^p < 0.001. Student’s *t*-test. – Coll: without collagen 1, + Coll: with collagen 1.

In order to determine whether the collagen 1 is involved in the survival of FCS-starved-MCF-7 and T-47D cells, we studied the cell mortality after seeding the cells (or not) on a 2D collagen 1 coating. We found a decrease in cell mortality in the presence of collagen 1 after 24 h and 48 h post-starvation with a greater effect at 48 h, when compared to plastic condition (Figure 1Aa-1Ab, N=3, *p*<0.001). Next, we analysed apoptosis in cells seeded or not on collagen 48 h post-starvation using double labelling Annexin V/IP. Figure 1B showed that apoptotic cell rate is reduced by collagen 1 in MCF-7 (23.1 ± 1.1% for control *vs* 13.93 ± 0.35% in the presence of collagen 1, N=3, *p*<0.001, Figure 1Ba), and T-47D cells (13.1 ± 0.5% for control *vs* 8.25 ± 0.05% in the presence of collagen 1, N=3, *p*<0.01, Figure 1Bb). Moreover, the collagen 1 reduced Bax expression level in both MCF-7 (70 ± 13.2%, N=3, *p*<0.01, Figure 1Ca) and T-47D cells (43.25 ± 5%, N=3, *p*<0.001, Figure 1Cb) without affecting that of Bcl2 (Figure 1Ca-1Cb). These data showed that in FCS-starvation condition, collagen 1 protected the cells against apoptosis.

### Collagen 1 promotes basal calcium influx and regulates cell survival through a calcium-dependent mechanism

**Figure 2:**
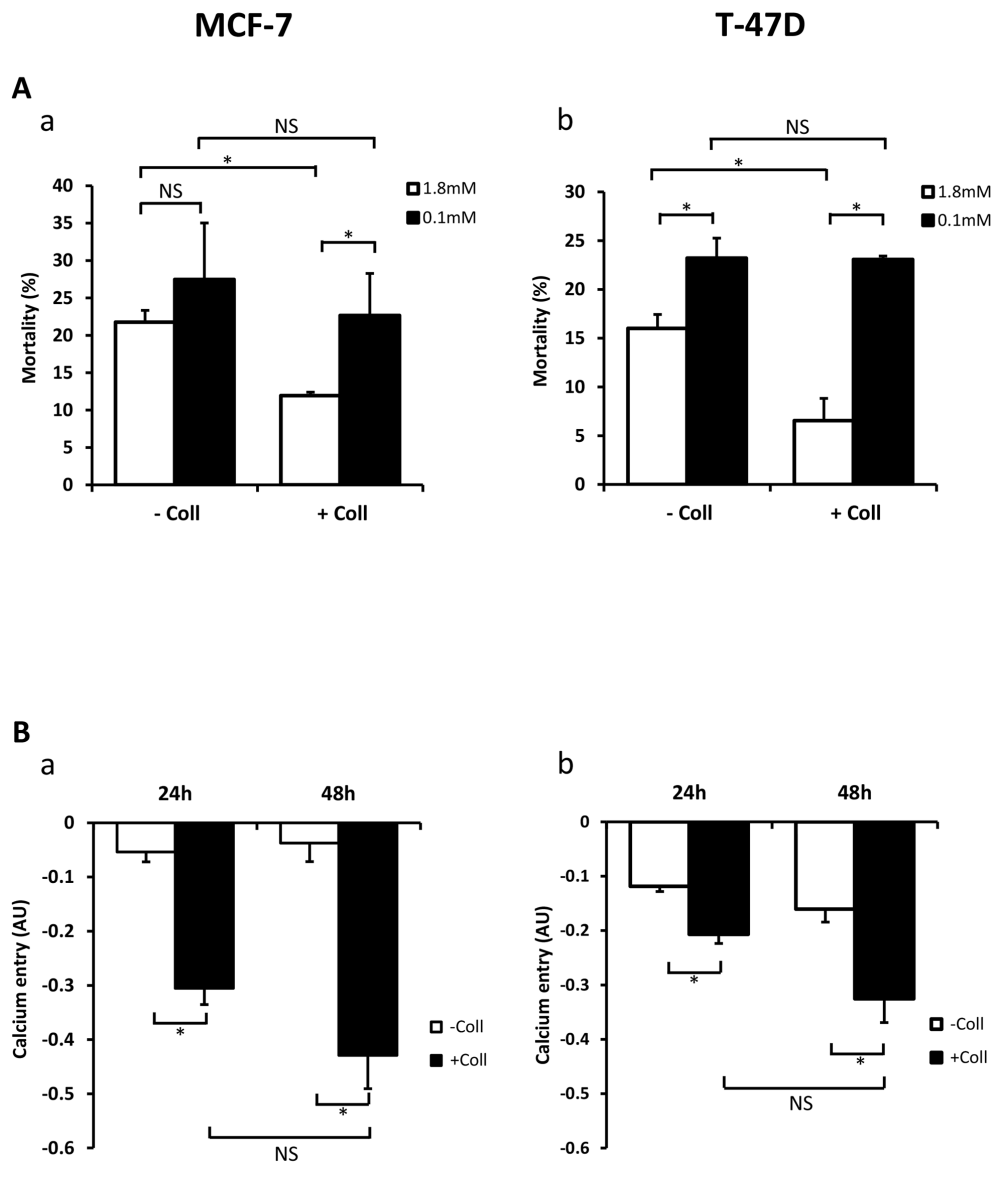
Collagen 1 promotes calcium influx and regulates cell survival by a calcium-dependent mechanism **(A)** Impact of decreasing extracellular Ca^2+^concentration on cell mortality of MCF-7 **(a)** and **(b)** T-47D cells. Mortality was measured 48 h post-starvation, with (+ Coll) or without collagen 1 (- Coll), after incubation with medium containing low (0.1 mM) or physiological Ca^2+^ concentrations (1.8 mM). Values are reported as mean ± SEM of triplicate experiments, ^*^p<0.05, NS: not significant. ANOVA followed by Holm-Sidak *post hoc* tests. **(B)** Effect of collagen 1 on basal Ca^2+^ entry in the same batch of MCF-7 (a) and T-47D (b) cells using Mn^2+^ quenching experiments. Mean slope values are reported as mean ± SEM of triplicate experiments, ^*^p<0.05, ANOVA followed by Holm-Sidak *post hoc* tests, NS: not significant.

We have already reported that extracellular Ca^2+^ regulates breast cancer cell survival and proliferation (Faouzi et al., 2011), we examined whether the variation of extracellular Ca^2+^ concentrations ([Ca^2+^]_o_) could affect the collagen-dependent survival. The cell viability was analysed after 48 h FCS-starvation. As expected, in 1.8 mM [Ca^2+^]_o_ (physiologic calcium condition) collagen 1 decreased cell mortality in MCF-7 and T-47D cells (Figure 2Aa-2Ab, N=3, *p*<0.05). However, it failed to decrease the cell mortality in low Ca^2+^ conditions (0.1 mM Ca^2+^) in both cell lines (Figure 2Aa-2Ab, N=3, *p*<0.05). Indeed, under low calcium conditions, collagen 1-induced cell mortality was increased compared to physiologic calcium conditions in both cell lines (Figure 2Aa-2Ab, N=3, *p*<0.05). Then, we investigated whether collagen 1 induced Ca^2+^ entry that we analysed through Mn^2+^ quenching of fura-2 fluorescence. Addition of Mn^2+^ decreased the fura-2 fluorescence in a time-dependent manner revealing a Mn^2+^ influx through plasma membrane Ca^2+^ channels [28]. Our results showed an increased Mn^2+^ quenching by 5.7 and 11.5 folds in MCF-7 and 1.74 and 2.02 folds in T47-D cells cultured on collagen 1 for 24 h and 48 h post-starvation respectively (Figure 2Ba-2Bb, N=3, *p*<0.05). Altogether, our results showed that the collagen 1 promotes BC cell survival through a Ca^2+^-dependent pathway.

### Collagen 1 increases Kv10.1 and Orai1 expressions and potentiates their co-localization

**Figure 3:**
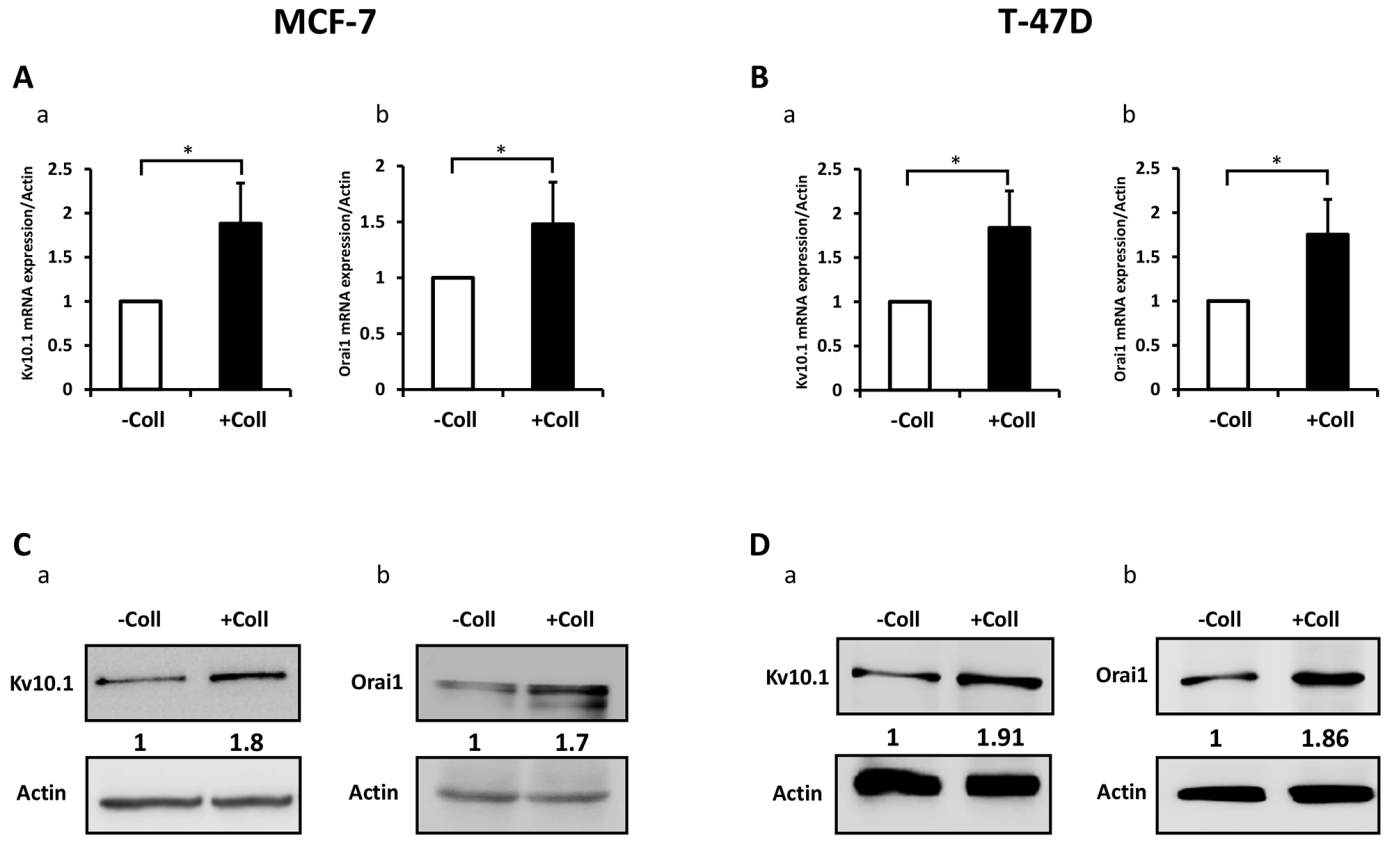
Collagen 1 increases Kv10.1 and Orai1 expression in both MCF-7 and T-47D cells **(A)** qRT-PCR expression of Kv10.1 **(a)** and Orai1 **(b)** in MCF-7 cells 48 h post-starvation in the presence of collagen 1. **(B)** qRT-PCR expression of Kv10.1 (a) and Orai1 (b) in T-47D cells 48 h post-starvation in the presence of collagen 1. **(C)** Representative western blots showing the expression of Kv10.1 (a) and Orai1 (b) in MCF-7 cells 48 h post-starvation in the presence of collagen 1. **(D)** Representative western blots showing the expression of Kv10.1 (a) and Orai1 (b) in T-47 cells 48 h post-starvation in collagen 1 condition. Results were normalized as a percentage of the control condition (- Coll). Values are reported as mean ± SEM of triplicate experiments. ^*^p <0.05, Student’s *t*-test.

**Figure 4:**
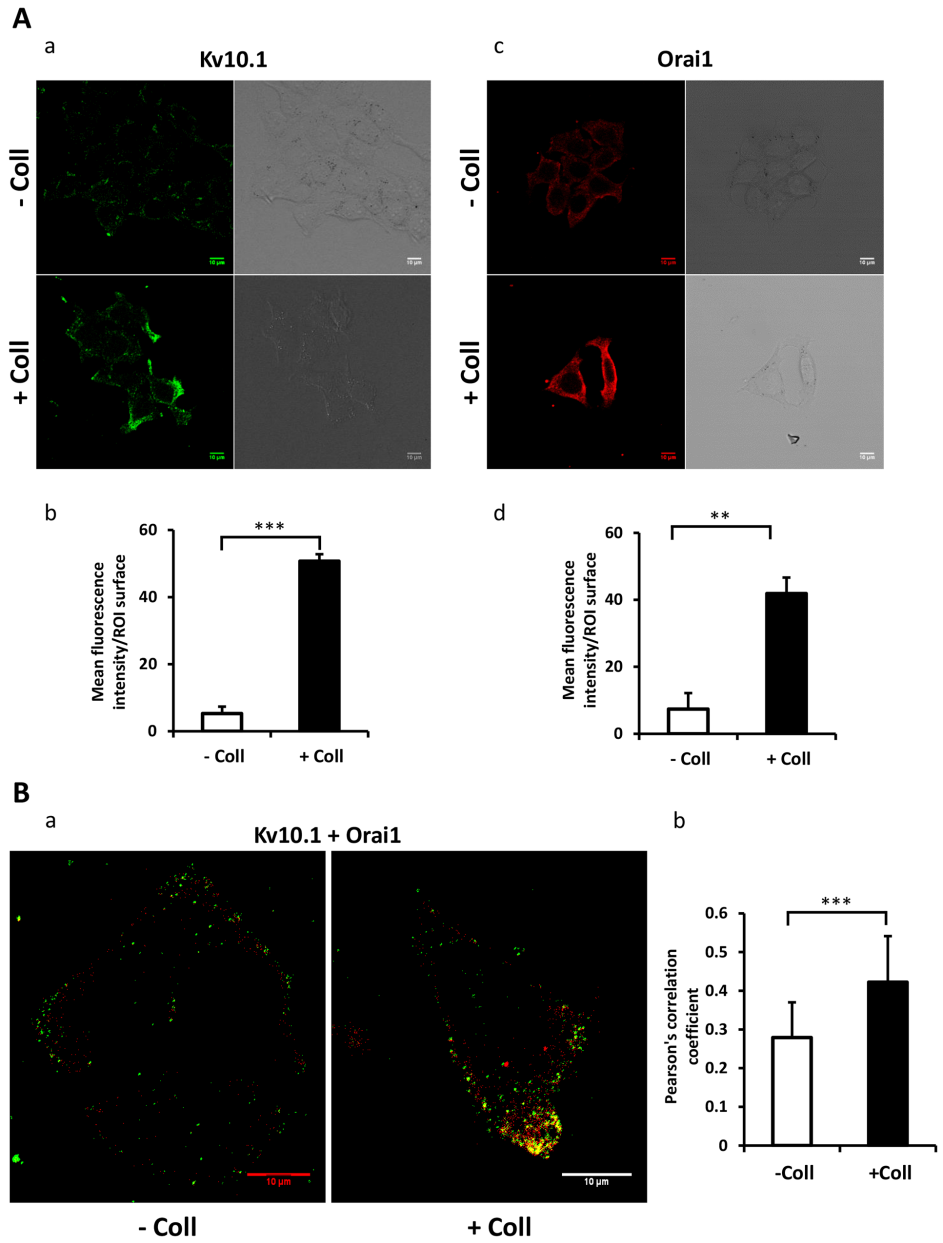
Collagen 1 promotes the co-localization of Kv10.1 and Orai 1 at plasma membrane in BC cells **(A)** Localization of Kv10.1 **(a)** and Orai1 **(c)** proteins at the plasma membrane 48 h post-starvation in MCF-7 cells seeded (+ Coll) or not (- Coll) on collagen 1, visualized by confocal microscopy. Quantification of the Kv10.1 **(b)** and Orai1 **(d)** fluorescence intensity in the two conditions. **(B)** Merge of Kv10.1 and Orai1 (yellow) at the plasma membrane 48 h post-starvation visualized by confocal microscopy (a), and Pearson’s correlation coefficient (b). Values are reported as mean ± SEM of triplicate experiments. ^**^p < 0.01, ^***^p < 0.001. Student’s *t*-test.

We have previously reported that Kv10.1 regulates cell migration in breast cancer cells by regulating basal calcium influx through Orai1 [26]. Here we investigated the effect of collagen 1 on Kv10.1 and Orai1 expressions. The expression of Orai1 and Kv10.1 was increased by collagen 1 at both mRNA and protein levels in both cell lines (Figure 3). mRNA of Kv10.1 and Orai1 were increased by collagen 1 in MCF-7 (1.8-fold for Kv10.1 and 1.5-fold for Orai1, Figure 3Aa-3Ab, N=3, *p*<0.05), and in T47-D cells (1.83-fold for Kv10.1 and 1.75-fold for Orai1, Figure 3Ba-3Bb, N=3, *p*<0.05). Western blotting experiments showed also an increase in the expression of Kv10.1 and the glycosylated form of Orai1 in both cell lines (Figure 3C-3D, N=3, *p*<0.01). Similar results were observed by confocal fluorescence microscopy (Figure 4Aa-4Ad, N=3, *p*<0.001 (Kv10.1), *p*<0.01 (Orai1)). In order to show a possible functional interaction between Kv10.1 and Orai1, we analysed Kv10.1 and Orai1 staining and their colocalization at the plasma membrane of MCF-7 cells by fluorescence microscopy. As shown in Figure 4Ba, Kv10.1 was co-localized with Orai1 only in the presence of collagen 1. Indeed, in the presence of collagen 1 the co-localization between these two channels reached 50% (Figure 4Bb, N=3, *p*<0.001). These data demonstrated that collagen 1 not only induced an increase in Kv10.1 and Orai1 expression but also potentiated their co-localization and interaction leading to the regulation of basal Ca^2+^ influx in BC cells.

### Kv10.1 and Orai1 are involved in collagen-dependent survival of BC cell lines

**Figure 5:**
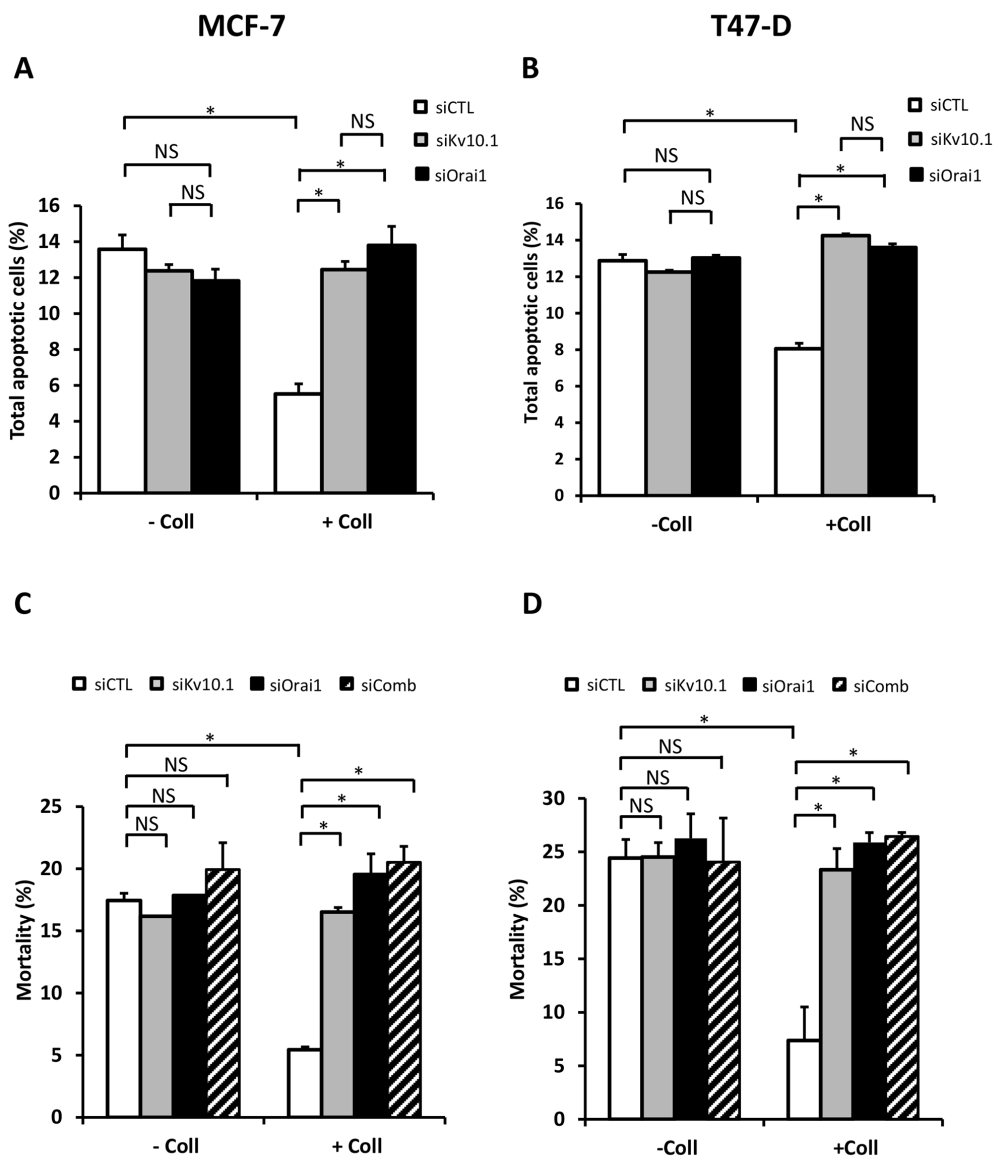
Kv10.1 and Orai1 are involved in collagen-dependent survival of BC cell lines Effect of Kv10.1 or Orai1 silencing on the apoptotic MCF-7 **(A)** and T-47D cells **(B)** rate. Cells were starved for 48 h and the apoptosis assay was carried out by annexin V/IP staining, values are reported as mean ± SEM of triplicate experiments, ^*^p<0.05, ANOVA followed by Holm-Sidak *post hoc* tests. **(C-D)** Effect of Kv10.1, Orai1 and Kv10.1 + Orai1 (siComb) silencing on MCF-7 **(C)** and T-47D **(D)** cell mortality. Cells were starved for 48 h and the mortality was measured by Trypan Blue assay, values are reported as mean ± SEM of triplicate experiments, ^*^p<0.05, ANOVA followed by Holm-Sidak *post hoc* tests.

To investigate if Kv10.1 and Orai1 contributed to the collagen-dependent survival, BC cells were transfected with anti-Kv10.1 and anti-Orai1 siRNAs independently and simultaneously. In both cell lines, treatment with anti-Kv10.1 siRNA (siKv10.1) reduced Kv10.1 expression in the absence (66.8 ± 0.12% in MCF-7 and 76.23 ± 15.5% in T-47D cells) and in the presence of collagen (65.12 ± 0.04% in MCF-7 and 73.83 ± 13.9% in T-47D cells) (Supplementary Figure 1A-1B, N=3, *p*<0.05). anti-Orai1 siRNA (siOrai1) was able to reduce Orai1 expression by 51.2 ± 17% in MCF-7 cells (51.86 ± 18% in T-47D cells) and by 53.18 ± 13% in MCF-7 cells (41.06 ± 16% in T-47D cells) in the same conditions respectively (supplementary Figure 1A-1B, N=3, *p*<0.05). These data showed that, whatever the experimental conditions (with or without collagen 1), the siRNA efficiency was similar. Moreover, neither Orai1, nor Kv10.1 regulated each other since silencing of one did not affect the expression level of the other. We then measured the effect of Kv10.1 and Orai1 silencing on apoptosis rate in MCF-7 and T-47D cells seeded or not on collagen 1. As expected in the presence of collagen 1, the siRNA control decreased apoptosis rate respectively by 58.47% and 37.48% in MCF-7 and T-47D cells when compared to plastic condition (Figure 5A-5B, *p*<0.05, N=3). The collagen-dependent survival disappeared when the cells were transfected with siKv10.1 or siOrai1. Indeed, the apoptosis rate of cells seeded on collagen (12.45 ± 0.45% for siKv10.1 and 13.8 ± 0.35% for siOrai1, N=3, in MCF-7 and 14.25 ± 0.1% for siKv10.1 and 13.6 ± 0.2% for siOrai1, N=3, in T-47D cells) was not significantly different from that observed in the absence of collagen 1 (11.85 ± 0.35% for siKv10.1 and 11.82 ± 0.64% for siOrai1, N=3 in MCF-7, and 12.25 ± 0.1% for siKv10.1 and 13.02 ± 0.15% for siOrai1, N=3 in T-47D cells) (Figure 5A–5B, *p*<0.05, N=3, Supplementary Figure 2A-2B). To examine if Kv10.1 and Orai1 shared similar signalling pathway, we analysed cell mortality in the presence of siRNA directed against both ion channels. Interestingly, no additive nor synergistic effects were observed in cells transfected simultaneously with siKv10.1 and siOrai1 when compared to the effects observed in the presence of each of the siRNAs alone (Figure 5C-5D, *p*<0.05, N=3).

**Figure 6:**
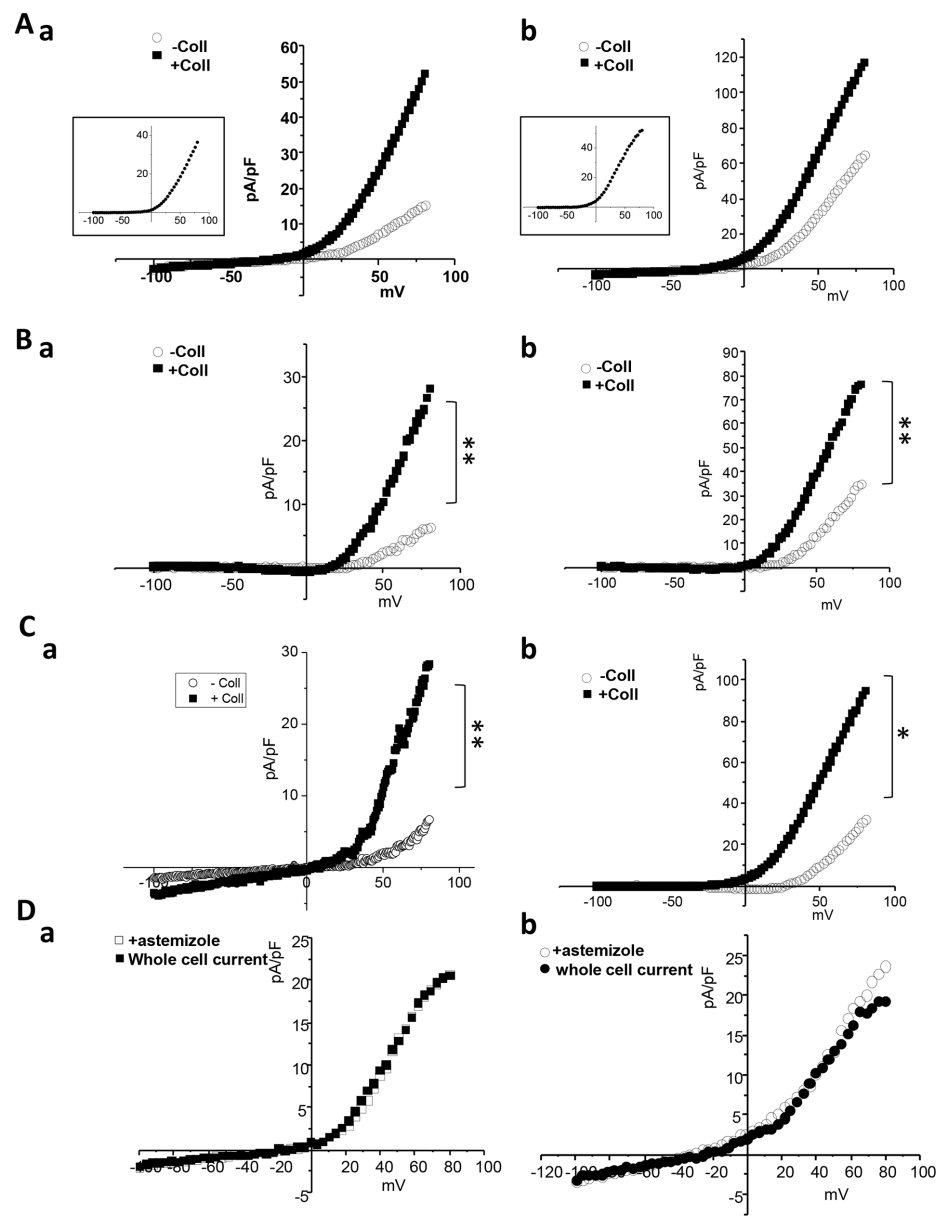
Collagen increases Kv10.1 functional channel activity in BC cells **(A)** Whole cell currents recorded in cells treated or not with collagen 1. Cells were starved for 48 h and the patch-clamp measurements were performed. 500 msec voltage ramps from -100 to +80 mV from a holding potential of -40 mV were applied to record Kv10.1 channel activity in MCF-7 **(a)** and T-47D **(b)** cell lines. The collagen-activated outward current is shown in the small squares. **(B)** Astemizole-sensitive current traces in MCF-7 (a) and T-47D (b) cells seeded or not on collagen 1 obtained from the subtraction of the current remained after astemizole perfusion from the whole cell current. **(C)** siKv10.1 sensitive current in MCF-7 (a) and T-47D (b) cells treated or not with collagen, obtained from the subtraction of the whole cell current recorded in cells transfected with siKv10.1 from the average whole cell current recorded in cells transfected with si-control. Values are reported as mean. ^*^p <0.05, ^**^p< 0.01. Mann-withney test. **(D)** Effect of the perfusion of astemizole (5 μM) on the outward current recorded in siKv10.1-tranfected MCF-7 (a) and T-47D (b) cells seeded on collagen 1. Astemizole failed to affect the amplitude of the remaining outward current in siKv10.1-transfected cells.

We also investigated the impact of collagen 1 on Kv10.1 activity. Both MCF-7 and T-47D cells show an increased outward current when treated with collagen 1 (Figure 6Aa-6Ab, MCF-7 cells: without collagen, 15.22 ± 2.28 pA/pF at 80 mV, n=5; with collagen, 51.66 ± 17.7 pA/pF, n=6, *p*<0.01. T-47D cells: without collagen, 64.47 ± 12.80 pA/pF at 80 mV, n=5; with collagen, 116.41 ± 19.92 pA/pF, n=5, *p*<0.01). Astemizole is classically used as a pharmcological inhibitor of Kv10.1 (Ouadid-Ahidouch et al., 2001); (Garcia-Ferreiro et al., 2004). We then perfused astemizole (10 μM) to isolate Kv10.1 mediated current. Both in MCF-7 and T-47D cells the astemizole sensitive-current was significantly higher in collagen treated cells (Figure 6Ba-6Bb, MCF-7 cells: without collagen, 5.88 ± 0.96 pA/pF at 80 mV, n=5; with collagen, 27.16 ± 9.94 pA/pF, n=6, *p<0.01*. T-47 D cells: without collagen, 34.86 ± 8.54 pA/pF at 80 mV, n=5; with collagen, 76.56 ± 12.80 pA/pF, n=5, *p<*0.01). To confirm this data we recorded whole cell currents in MCF-7 and T-47D cells in which Kv10.1 was silenced. Also the current obtained from the subtraction of siKv10.1 cells current and the control whole cell current was higher in collagen treated cells (Figure 6Ca-6Cb, MCF-7 cells: without collagen, 6.9 ± 4.52 pA/pF at 80 mV; with collagen, 29.02 ± 8.76 pA/pF, n=6, *p<0.01*. T-47D cells: without collagen, 31.98 ± 7.86 pA/pF at 80 mV; with collagen, 94.02 ± 9.82 pA/pF, n=5, *p<*0.05). In all cases the difference between the amplitude of astemizole- and siKv10.1- sensitive currents was not significant (Figure 6Da-6Ab), confirming the inhibitory effect of the drug on Kv10.1 channel.

### Both Kv10.1 and Orai1 are involved in collagen-dependent calcium influx in BC cells

**Figure 7:**
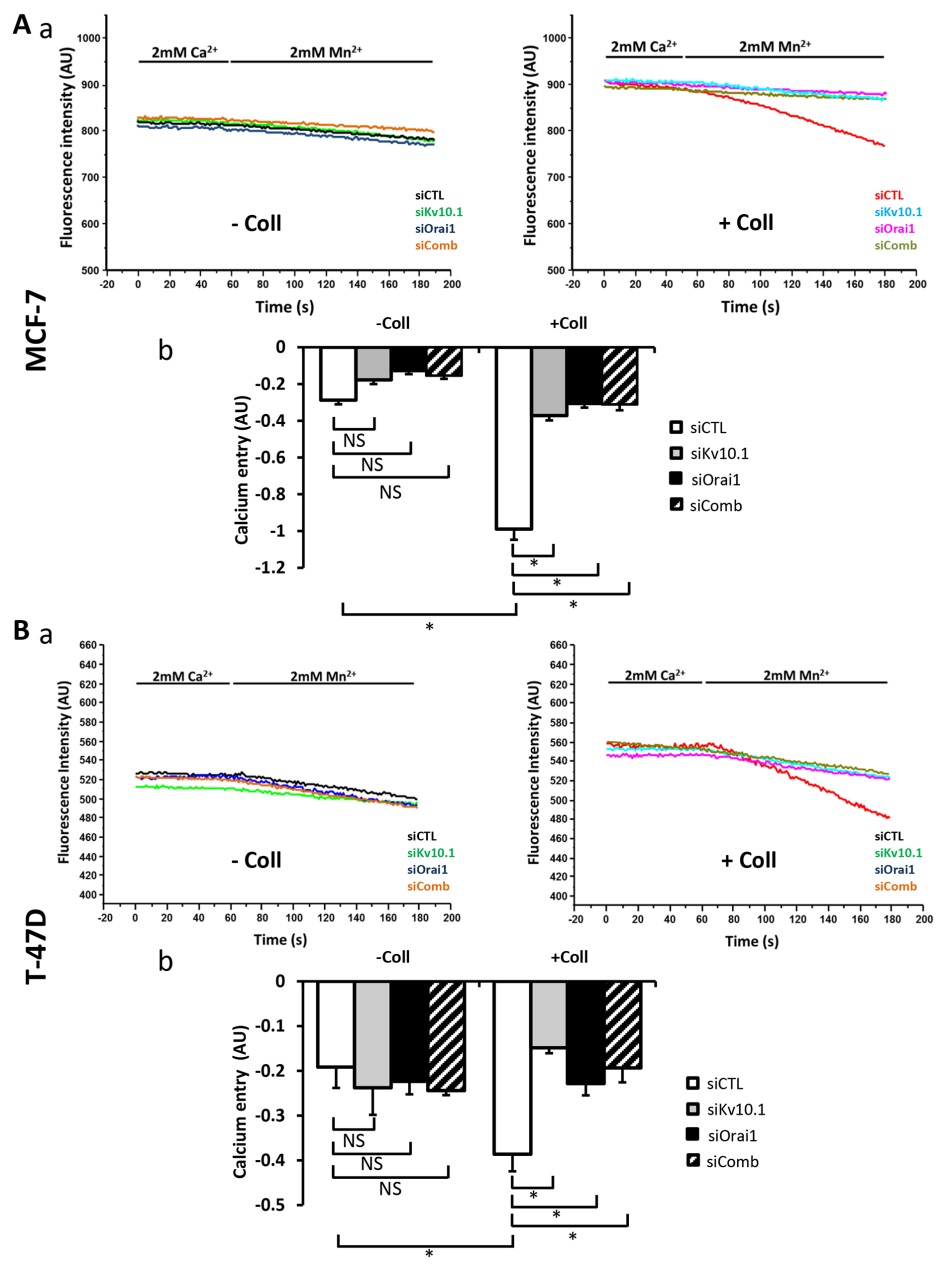
Kv10.1 regulates collagen 1-calcium entry through Orai1 **(A)** Effect of Kv10.1 and Orai1 and Kv10.1 + Orai1 (siComb) silencing on Ca^2+^ entry in MCF-7 cells, by using Mn^2+^ quenching experiments **(a)**. Mean slope values are reported as mean ± SEM of triplicate experiments performed on 5 different number of cell passage **(b)**, ^*^p<0.05, ANOVA followed by Holm-Sidak *post hoc* tests. **(B)** Effect of Kv10.1, Orai1 and kv10.1 + Orai1 (siComb) silencing on Ca^2+^ entry in T-47D cells, by using Mn^2+^ quenching experiments (a). Mean slope values are reported as mean ± SEM of triplicate experiments performed on 3 different number of cell passage (b), ^*^p<0.05, ANOVA followed by Holm-Sidak *post hoc* tests.

To determine whether Kv10.1 and Orai1 regulated Ca^2+^ entry induced by collagen 1, we performed Mn^2+^ quenching analysis. In both cell lines, siKv10.1 and siOrai1 inhibited the collagen dependent Ca^2+^ influx by 62 ± 0.22% (N=3, *p*<0.05), and 68 ± 4.02% (N=3, *p*<0.05) respectively in MCF-7 cells, and by 60.89 ± 4.33% (N=3, *p*<0.05) and 39.86 ± 6.67% (N=3, *p*<0.05) respectively in T-47D cells when compared to the control siRNA (Figure 7A-7B). Moreover, no additive effect was observed when cells were simultaneously treated with both siRNAs (68.5 ± 2.93% and 49.03 ± 5.6% of inhibition in MCF-7 and T-47D cells respectively (Figure 7A–7B, *p*<0.05, N=3). We also investigated the effect of anti-Kv10.1 and anti-Orai1 siRNAs on Ca^2+^ current recorded in MCF-7 cells (Supplementary Figure 3Aa-3Ac). Patch clamp whole cell recordings showed a significant increase in Ca^2+^ inward current induced by collagen 1 at -100 mV (without collagen 1: -2.75 ± 0.23 pA/pF, with collagen 1: -3.87 ± 0.26 pA/pF, n=7, *p*<0.05). This increase was significantly reduced after the silencing of either of Orai1 (-2.28 ± 0.35 pA/pF, n=5, *p*<0.01) or Kv10.1 (-2.93 ± 0.26 pA/pF, n=7, *p*<0.05). However, Orai1 or Kv10.1 silencing had no effect on Ca^2+^ current in plastic conditions (Supplementary Figure 3Ab-3Ac). We also performed experiments on cell mortality and calcium entry using Kv10.1 pharmacological inhibitor. Treatment by astemizole (5 μM) led to an increase of cell mortality and a decrease of Ca^2+^ entry of cells seeded or not on collagen 1 (Supplementary Figure 3B-3C, *p*<0.05, N=3). In non-treated cells, the rate of mortality decreased in the presence of collagen 1. However, when the cells were treated with astemizole, the rate of mortality increased and reached a level similar to that observed when the cells were seeded on plastic (Supplementary Figure 3B, N=3, *p*<0.05). Moreover, astemizole completely suppressed the collagen-dependent Ca^2+^ entry (Supplementary Figure 3C, N=3, *p*<0.05).

### Collagen 1 overexpressed Kv10.1 and Orai1 through ERK1/2 but not Akt pathway

**Figure 8:**
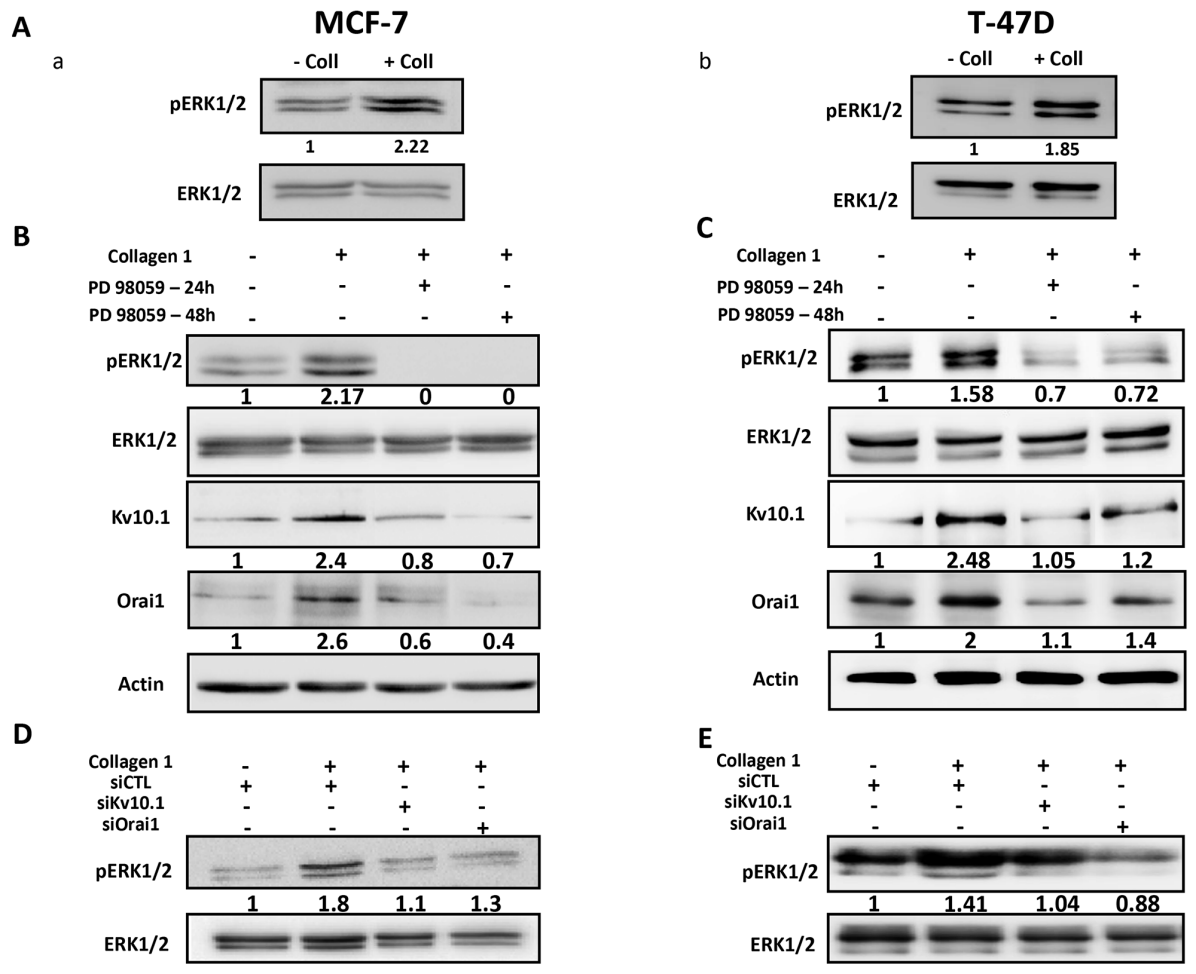
Collagen 1 increases Kv10.1 and Orai1 expression through ERK1/2 pathway **(A)** Representative western blots showing ERK1/2 phosphorylation in MCF-7 (a) and T-47D (b) cells 48 h post-starvation in presence (+ Coll) and absence (- Coll) of collagen 1. **(B-C)** Representative western blots showing the effect of PD98059 (40 μM) on ERK1/2 activation, and on Kv10.1 and Orai1 expression in MCF-7 **(B)** and T-47D **(C)** cells.Values were normalized as a percentage of the control condition (- Coll). **(D-E)** Representative western blots showing the effect of Kv10.1 and Orai1 silencing on ERK1/2 phosphorylation in MCF-7 **(D)** and T-47D **(E)** cells in collagen 1 conditions. – Coll: without collagen 1, + Coll: with collagen 1.

Several studies have reported the activation of ERK and Akt pathways in cell survival in the presence of collagen 1 [29, 6]. We therefore investigated whether these pathways were regulated by collagen 1 in our models. Cells seeded on collagen 1 coating showed an increase in ERK1/2 phosphorylation in the absence of FCS when compared to their counterparts seeded on plastic (2.27 ± 0.4 and 1.61 ± 0.15 fold for MCF-7 and T-47D cells respectively (Figure 8Aa-8Ab, N=3-5, *p*<0.001 (MCF-7), *p*<0.05 (T-47D)). However, collagen 1, in the same conditions, failed to activate Akt (Supplementary Figure 4Aa-4Ab, N=3, *p*>0.05). In order to investigate whether ERK1/2 was involved in the collagen-dependent overexpression of Kv10.1 and Orai1, cells were seeded on collagen 1 coating and treated with the ERK1/2 inhibitor PD98059 (40 μM) during 24 h and 48 h. Western blot analysis in Figure 8B-8C showed that PD98059 treatment abolished the collagen-dependent overexpression of Kv10.1 and Orai1 in both cell lines. In fact, Kv10.1 and Orai1 expression was reduced respectively by 62.46 ± 5.02% and 62.73 ± 15.9% after 24 h treatment, and by 64.13 ± 6.4% and 73.3 ± 11.6% after 48 h treatment by PD98059 in MCF-7 cells (N=3, *p*<0.05) and by 60.17 ± 17% and 58.65 ± 3% after 24 h treatment, and by 70.07 ± 20.4% and 56.88 ± 16.88 % after 48 h treatment in T-47D cells (N=3, *p*<0.01). Moreover, treatment with PD98059 during 24 h and 48 h completely suppressed the collagen-dependent survival effect in both MCF-7 and T-47D cells (Supplementary Figure 4Ba-4Bb, N=3, *p*<0.05).

It is well known that ERK1/2 activity is under regulation of Ca^2+^ [30, 31] and K^+^ channels [32]. We therefore evaluated the effect of siKv10.1 and siOrai1 on ERK1/2 phosphorylation. Figure 8 D-E showed that Kv10.1 and Orai1 silencing inhibited the ERK1/2 activation in MCF-7 and T-47D cells (Figure 8D-8E, N=3, *p*<0.05). Taken together, these data suggested that collagen 1, by activating ERK1/2 pathway, induced an overexpression of Kv10.1 and Orai1 ion channels leading to Ca^2+^ influx that, in turn, permited maintained ERK1/2 activation allowing therefore the protection of the cells against apoptosis.

### Collagen-dependent survival, through Kv10.1 and Orai1 overexpression, is promoted by DDR1

**Figure 9:**
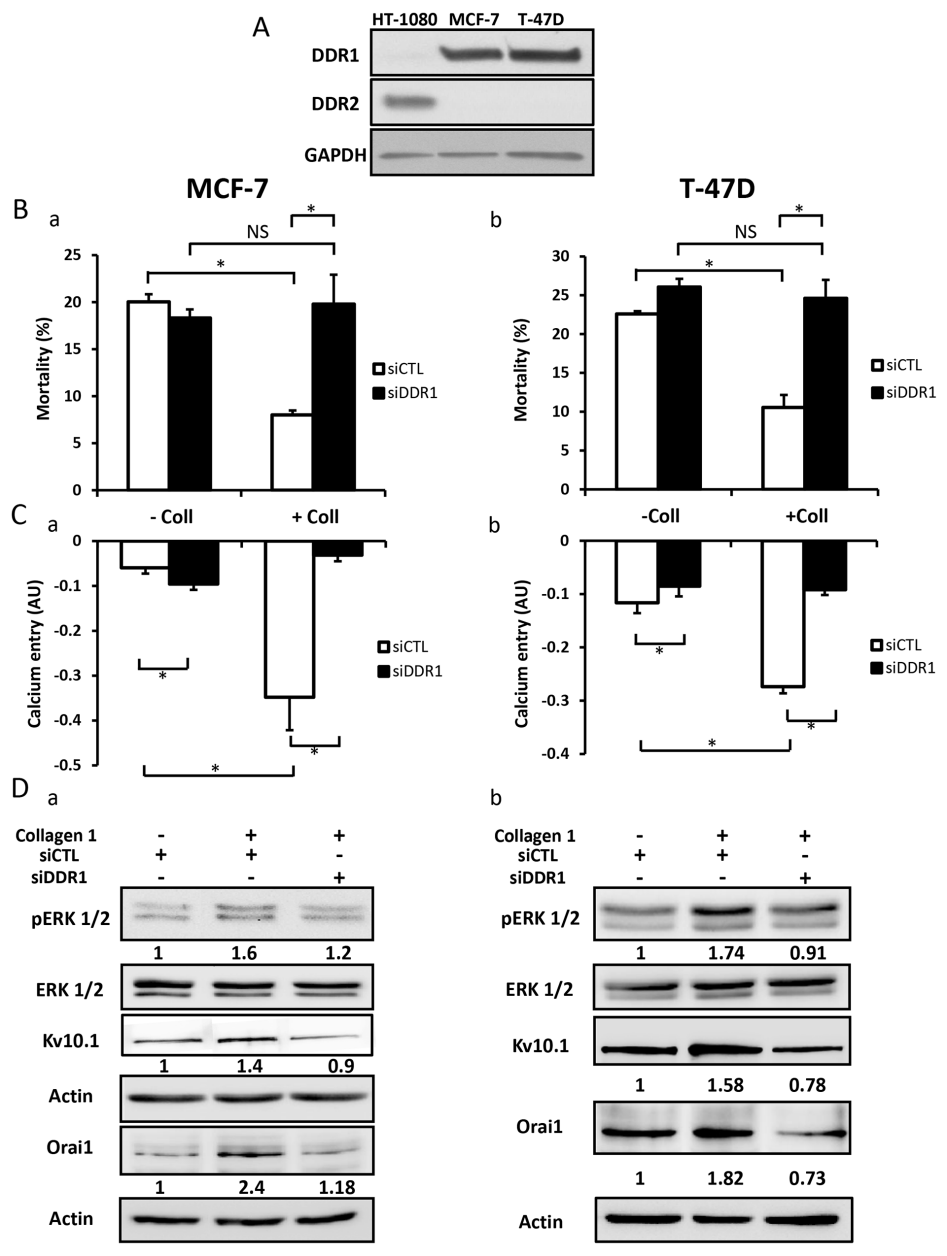
DDR1 is expressed in MCF-7 and T-47D cells and promoted collagen-dependent survival **(A)** Representative western blot showing DDR1 and DDR2 expression in MCF-7 and T-47D cells. **(B)** Effect of DDR1 silencing on cell mortality of MCF-7 (a) and T-47D cells (b). Cells were starved for 48 h and the mortality was measured by Trypan Blue assay, values are reported as mean ± SEM of triplicate experiments performed on 3 different number of cell passage, ^*^p<0.05, ANOVA followed by Holm-Sidak *post hoc* tests. **(C)** Effect of DDR1 silencing on Ca^2+^ entry in MCF-7 (a) and T-47D (b) cells. Mean slope values are reported as mean ± SEM of triplicate experiments performed on 4 different number of cell passage, ^*^p<0.05, ANOVA followed by Holm-Sidak *post hoc* tests. **(D)** Representative western blot showing the effect of DDR1 silencing on ERK1/2 phosphorylation, Kv10.1 and Orai1 expression in MCF-7 (a) and T-47D (b) cells seeded on collagen 1.

DDR1 and DDR2 are tyrosine kinase receptors which are activated by collagen 1 [33, 34]. Collagen binding to DDRs triggers several downstream signalling pathways including ERK1/2 MAP kinase and PI3-kinase-AKT [35]. We first investigated which type of DDRs was expressed in BC cells, and investigated its involvement in the overexpression of Orai1 and Kv10.1, as well as in ERK1/2 phosphorylation and cell survival. Only DDR1, and not DDR2, was expressed in MCF-7 and T-47D cells (Figure 9A, N=3). DDR1 silencing completely abolished the collagen-dependent cell survival (Figure 9Ba-9Bb, N=3, *p*<0.05) and reduced the basal Ca^2+^entry (Figure 9Ca-9Cb, N=3, *p*<0.05). The validations of siRNA targeting DDR1 in BC cells are shown in Supplementary Figure 5A. Furthermore, collagen 1 failed to affect the expression of Kv10.1, Orai1, or ERK1/2 activation in both MCF-7 and T-47D cells transfected with siRNA anti-DDR1 (Figure 9Da-9Db, N=3, *p*<0.05). These data suggested DDR1 as a potential candidate interacting with collagen 1 to induce intracellular cell signalling that activates ERK1/2 and consequently induce an increase in Kv10.1 and Orai1 ion channel expression. This process likely increased cell survival in FCS deprivation conditions.

β1-integrin is also able to bind collagen 1. To check this hypothesis, we investigated the impact of silencing β1-integrin on DDR1 expression, cell mortality, and calcium entry in MCF-7 cells. Data show that silencing of β1-integrin failed to affect DDR1 expression, apoptotis rate and calcium entry when cells were seeded on collagen 1 coating (Supplementary Figure 5B-5D, N=3, *p*>0.05).

## DISCUSSION

Our study demonstrates, for the first time, a new pathway involving tumour microenvironment which regulates Kv10.1 expression and function, and consequently cell survival in breast carcinoma. We found that collagen 1 coating is associated with an increase in Kv10.1 and Orai1 channels expression and activity, and basal Ca^2+^ entry. This event activates ERK1/2 pathway, downregulates Bax expression, and increases survival of FCS-starved BC cells. Moreover, collagen 1 promotes the co-localisation of Kv10.1 with Orai1 at the plasma membrane.

Breast cancer cell proliferation and survival are dependent on both K^+^ and Ca^2+^ ions [24, 36, 37, 38, 39]. Indeed, in 2D plastic culture, without extracellular matrix (ECM) coating, pharmacological or downregulation of K^+^ channels inhibits cell proliferation and induces a G1 phase arrest in the cell cycle. Moreover, cytosolic Ca^2+^ concentration ([Ca^2+^]_i_) is reduced [24]. Among K^+^ channels, Kv10.1 drives MCF-7 cells into G1 phase by hyperpolarizing the resting membrane potential leading to an increase of [Ca^2+^]_i_. Furthermore, the decrease in the extracellular Ca^2+^ concentration reduces [Ca^2+^]_i_, inhibits MCF-7 cell proliferation and increases apoptosis suggesting that Ca^2+^ influx is also essential for cell proliferation and survival [36]. Recently, a functional coupling between Kv10.1 and Orai1 channels has been reported in MDA-MB-231 breast carcinoma cells [26]. Orai1 is also reported to regulate cell proliferation of MCF-7 and T-47D cells [39]. Although the role of Kv10.1 and Orai1 are extensively studied in proliferation and migration of breast carcinoma cells [39, 40, 25, 26, 41], little is known about the molecular mechanism(s) underlying their role in cell survival.

Our results show that FCS-starved BC cells displayed a high survival rate when seeded on collagen 1 coating compared to the plastic condition. This effect is associated with an increase in Kv10.1 expression at both mRNA and protein levels. In agreement with these data, Toral *et al.* showed a high proliferation rate and a low mortality level in CHO cells stably overexpressing Kv10.1 and seeded on collagen 1 coating [27]. In one of our previous works, we showed that Kv10.1 by regulating the resting membrane potential promotes basal calcium entry through Orai1 which is necessary for cell migration [26]. In agreement with these data, we show in the present work that collagen 1 also increased Orai1 expression and Ca^2+^ influx. Using confocal imaging, we observed that collagen 1 potentiates both channels localization at the plasma membrane and promoted their co-localization. This suggests that collagen 1 is required for a physical interaction between the two channels which will be necessary to promote cell survival. In fact, the collagen-mediated survival was dependent on extracellular Ca^2+^. The levels of Ca^2+^ influx were significantly higher when the cells were seeded on collagen coating (Figure 2). Moreover, the reduction of [Ca^2+^]_o_ abolished the collagen 1 effect. This is in agreement with data reported by Girault *et al.* showing that coating alveolar epithelial cells with fibronectin, another ECM element, increases [Ca^2+^]_i_ probably through a Ca^2+^ influx via TRPC4 [42]. Moreover, collagen-mediated survival and calcium influx were abolished in the cells transfected with siKv10.1 or siOrai1 or when inhibiting pharmacologically Kv10.1 activity. Interestingly, the simultaneous downregulation of Kv10.1 and Orai1 expression showed that there was no additive effect on Ca^2+^ influx and cell survival, confirming that the Kv10.1/Orai1 complex works in a sequential manner and is a key player in the cross-talk between collagen 1 and breast carcinoma cells. The link between Kv10.1 and Orai1 seemed to be specific. Indeed, collagen 1 increases the expression of Orai3; which is involved in MCF-7 cell survival and proliferation (Faouzi et al., 2011). However, silencing Orai3 did not affect cell survival, proliferation or Ca^2+^ influx induced by collagen 1 (Supplementary Figure 6).

One of the interesting findings in this report is the identification of a loop between ERK1/2 activation and Kv10.1 and Orai1 channels. On one hand, we identified a functional role of the MAP/ERK1/2 pathway in the upregulation of Kv10.1, Orai1 and the collagen 1-induced cell survival. Cell treatment with the ERK1/2 inhibitor PD98059 completely abolished the overexpression of Kv10.1, Orai1 and the cell survival. On the other hand, we found that ERK1/2 phosphorylation was regulated by Kv10.1 and Orai1. Indeed, in collagen-treated cells, silencing Kv10.1 or Orai1 suppressed ERK1/2 activity. Several studies have shown that Ca^2+^ is highly involved in the phosphorylation of ERK1/2 [31, 43], but few studies have associated Orai1 with ERK1/2 phosphorylation [30, 41]. Our data suggest that collagen 1, by activating ERK1/2 potentiates the co-localization of Kv10.1 and Orai1 at plasma membrane allowing calcium influx that leads to maintain ERK1/2 activation and cell survival. We have previously demonstrated that the transcription factor c-Myc participates to MCF-7 survival. C-Myc is under ERK1/2 activation [43]. Our observation that collagen 1 increased c-Myc expression but not Fos or Jun expression suggested a potential involvement of c-Myc in the collagen 1-survival (Supplementary Figure 7).

**Figure 10:**
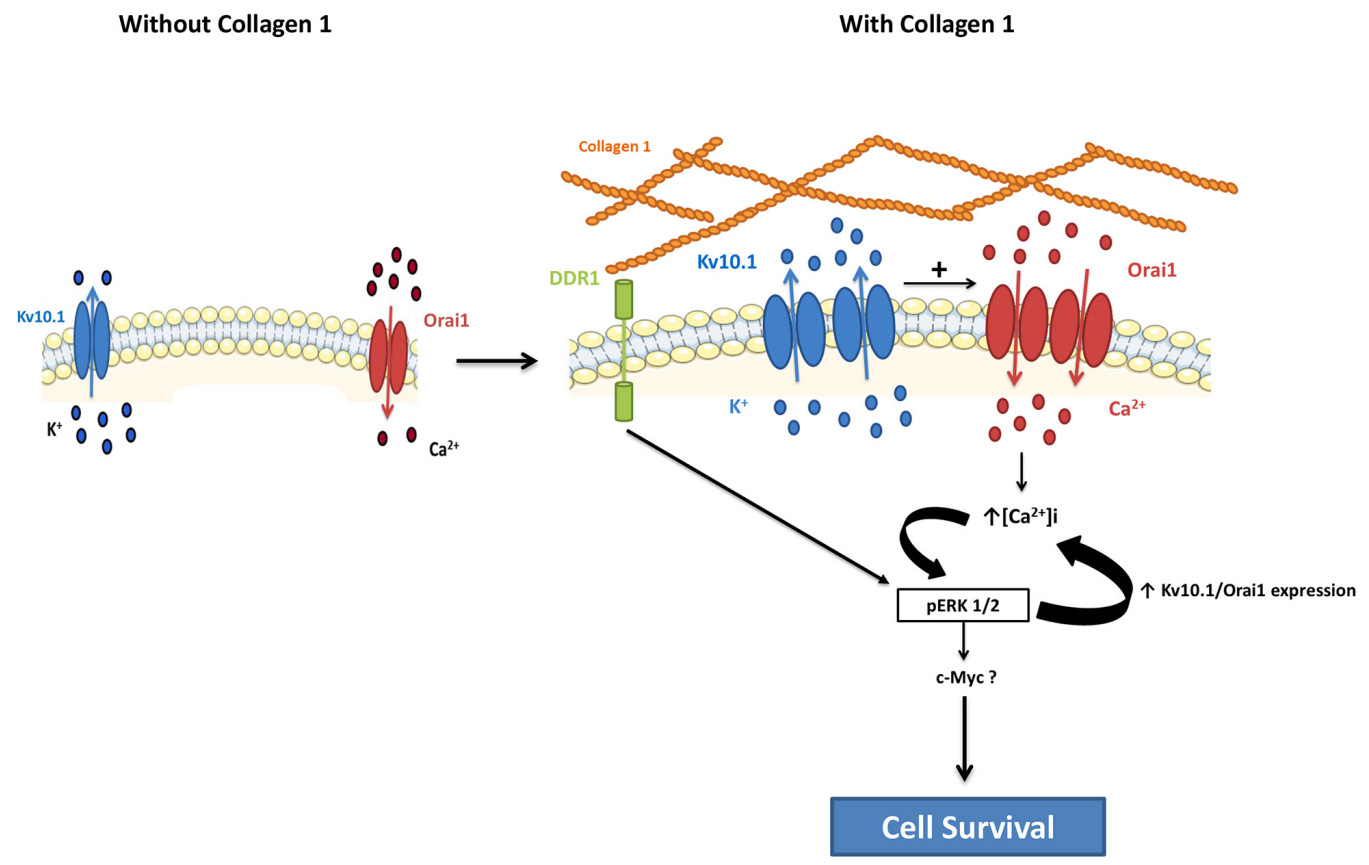
Schematic representation of potential involvement of the collagen 1 in breast cancer cell survival

The observation that Kv10.1 and Orai1 complex function in cell survival was dependent on the presence of collagen 1 suggested that a potential collagen receptor was involved in the activation of a signalling pathway leading to the observed effects. Our data showed that silencing of DDR1 completely abolished collagen-induced overexpression of Kv10.1 and Orai1, Ca^2+^ influx and the increase in cell survival. DDR1 has been described as one of the major tyrosine kinase receptors able to be activated by several collagens, including collagen 1 [12], and has been shown to be overexpressed and associated with bad prognosis in several cancer pathologies [44, 45, 46]. DDR1 promotes both Notch-1 [47] and NFκB activation [48]. We show an activation of the ERK1/2 pathway only when the BC cells expressing DDR1 were seeded on collagen 1. It’s important to note that our data are not in agreement with those reported recently by [13]. By using collagen tridimensional culture and without FCS-starvation, the authors have shown that collagen is able to activate DDR1 in MCF-7 cells and to induce apoptosis as revealed by the overexpression of the apoptosis marker BIK. In our model, two-dimensional collagen coating was used and the cells were FCS-starved. Like DDR1, c-Met is a well-known membrane receptor which is able to induce survival as well as apoptosis through different mechanisms. These divergent effects depend on the experimental model and stress conditions [49]. Finally, we demonstrate that DDR1 is involved in the Kv10.1-dependence of collagen 1 induced survival in FCS starvation condition. Transfection of MCF-7 and T-47D cells with siRNA anti-DDR1 significantly reduced the expression of Kv10.1, Orai1, Ca^2+^ influx and completely suppressed the cell survival in the presence of collagen 1. It is known that collagen can also bind to integrin that activates ERK1/2 cascade contributing to survival signalling [6, 50]. We found that silencing of β1-integrin failed to affect the expression of DDR1 receptor, cell survival and Ca^2+^ entry induced by collagen 1. Furthermore, no association between Kv10.1 channels and β1-integrin has been reported in CHO cells stably expressing Kv10.1 [27]. Taken together, our data suggest that in MCF-7 and T-47D breast carcinoma cells, when seeded on collagen 1, DDR1 but not β1-integrin activates ERK1/2 promoting Kv10.1 and Orai1 overexpression leading to persistent MAPK signalling activation. This might contribute to c-Myc expression and/or activity leading to a decrease in Bax expression and an increase in cell survival (Figure 10). Taking into account the importance of collagen 1 as the major component of the microenvironment in BC progression, Kv10.1 overexpression, in association at least with Orai1, constitutes a potential checkpoint of tumor progression positively regulated by the tumor microenvironment.

## MATERIALS AND METHODS

### Cell culture

MCF-7, T47-D and HT1080 cells were cultured in Eagle’s minimum essential medium (EMEM, Life Technologies, Saint-Aubin, France) supplemented with 5% Fetal Bovine Serum (FBS), 2 mM L-glutamine and 0.06% HEPES. Cultures were maintained at 37°C in a humidified atmosphere containing 5% CO_2_. Cells were weekly trypsinised using Trypsin-EDTA and screened for the presence of mycoplasma.

### Extraction and preparation of collagen 1

Collagen 1 had been extracted from rat tail tendons as recently described (Saby et al., 2016). For experiments requiring coating with collagen, each well was coated by adding 0.5 mL of nonpepsinized collagen 1 solubilized in 0.018 M acetic acid at a concentration of 35 μg/mL. Then, coated substrates were dried at room temperature under sterile conditions during one night. Therefore, wells were washed twice with PBS (Sigma Aldrich, Saint-Quentin-Fallavier, France) in order to eliminate completely acetic acid and the cells were plated in each well in the presence of complete MEM culture medium at a pH value of 7.20 – 7.40.

### Cell transfection and RNA interference

Transfection of cells was performed using nucleofection technology (Amaxa Biosystems, Lonza, Aubergenville, France) according to the protocol as previously described [43]. Cells were transiently transfected with siRNA directed against Kv10.1 (Dharmacon Research, Chicago, IL), Orai1 (Dharmacon Research, Chicago, IL), DDR1 (Santa Cruz Biotechnology, Inc., Heidelberg, Germany) and β1-integrin (Santa Cruz Biotechnology, Inc., Heidelberg, Germany), or with scrambled siRNA as a control (siCTL) (siGENOME Non-Targeting siRNA, Dharmacon Research, Chicago, IL), and used 72 h after transfection.

### Cell mortality

Cells were grown in Petri-dishes coated or not with 2D collagen 1. The cell mortality was assessed by Trypan blue assay. MCF-7 and T-47D cells were grown in 35 mm Petri-dishes at a density of 5×10^4^ or 10 ×10^4^ cells, after 24 h, cells were starved. 48 h post-starvation cells were removed by trypsinization and diluted in Trypan Blue (Sigma Aldrich, Saint-Quentin-Fallavier, France). Cell counts were performed six times (in a blind manner) and the results were expressed as the percentage of dead cells using the formula: rate of cell death = number of dead cells/number of total cells × 100.

### Western blotting

Cell lysates were extracted with RIPA buffer (1% triton x 100, 1% Na-deoxycholate, 150 mM NaCl, 10 mM PO4Na2/K, pH=7.2) supplemented with protease inhibitor cocktail (Sigma Aldrich, Saint-Quentin-Fallavier, France). Protein concentrations were measured using BCA method (Bio-Rad, Marnes-La-Coquette, France). Proteins were separated by denaturing SDS–PAGE and transferred onto nitrocellulose membranes (Hybond, GE Healthcare, Saclay, France). The primary antibodies used were: anti-Kv10.1 (1:200, Santa Cruz Biotechnology, Inc., Heidelberg, Germany), anti-Orai1 (1:200, Sigma Aldrich, Saint-Quentin-Fallavier, France), anti-Bcl2 (1:500, Cell Signalling Tech., Danvers, USA), anti-ERK1/2 (1:500, Cell Signalling Tech., Danvers, USA), anti-p-ERK1/2 Thr202/Tyr204(1:500, Cell Signalling Tech., Danvers, USA), anti-DDR1 (1:500, Cell Signalling Tech., Danvers, USA), anti-Bax (1:1,000, BD Biosciences, CA, USA), anti-DDR2 (1:2,000, R&D systems, Lille, France), anti-Akt (1:500, Cell Signalling Tech., Danvers, USA), anti p-Akt Ser473 (1:500, Cell Signalling Tech., Danvers, USA) and anti-β1-integrin (1:500, Cell Signalling Tech., Danvers, USA). Antibodies are followed by secondary antibodies coupled to horseradish peroxidase. Actin (1:3,500 Santa Cruz Biotechnology, Inc., Heidelberg, Germany) and GAPDH (1:1,000, Cell Signalling Tech., Danvers, USA) antibodies was used for loading control experiments. Bands were detected using an enhanced chemiluminescence kit (GE Healthcare, Saclay, France) and quantified using the densitometric analysis option in the Bio-Rad image acquisition system (Bio-Rad Laboratories, Marnes-la-Coquette, France).

### qRT -PCR

Total RNA was extracted via the standard Trizol-Phenol-Chloroform protocol. The concentration and the purity of the total RNA were determined by using a spectrophotometer (NanoDrop 2000, Wilmington, USA). The cDNA was synthesized from 1 μg of total RNA extracted with random hexamers and MultiScribe™ ReverseTranscriptase (Applied Biosystems, Carlsbad, USA). For the real time PCR, sens and antisens PCR primers specific to Kv10.1 (forward 5’ CGCATGAACTACCTGAAGAC-3’ and reverse 5’- TCTGTGGATGGGGCGATGTTC-3’), Orai1 (forward 5’-AGGTGATCAGCCTCAACGAC-3’ and reverse 5’- CGTATCATGAGCGCAAACAG-3’), c-Myc (forward 5’-CTCCTCACAGCCCACTGGTC-3’ and reverse 5’- CTTGGCAGCAGGATAGTCCTTC-3’) and β Actin (forward 5’-CAGAGCAAGAGAGGCATCCT-3’ and reverse (5’-ACGTACATGGCTGGGGTG-3’) were used. Real Time PCR was performed on a LightCycler System (Roche, Basel, Switzerland) using a mix containing SYBR green (Applied Biosystems, Carlsbad, CA). Kv10.1, Orai1 and c-Myc mRNA expression were normalized to the endogenous control (β-actin) and compared to the reference sample (without collagen) using the Pfaffl method [51].

### Kv10.1 and Orai1 staining

MCF-7 cells were gently and briefly washed with buffer containing 1 mM MgCl2, 100 mM KCl and 20 mM HEPES at 37°C. Then the cells were fixed and permeabilizated using 4% paraformaldehyde in PBS supplemented with 40 μg/mL digitonin for 10 min. Before staining, cells were washed three times with PBS and treated for 45 min with 10% normal goat serum (NGS) at room temperature for non-specific sites saturation. The anti-Kv10.1 (Alomone Labs, Jerusalem, Israel) and the anti-Orai 1 (ProSci Inc, CA, USA) antibodies were added at 1:100 dilution for 1 hour. Then, after a second NGS treatment, a secondary AlexaFluor^®^ 633 conjugate antibody was used at 1:100 dilution for Orai1 detection (Invitrogen, Cergy-pontoise, France). For Kv10.1 detection, a biotinylated anti-goat secondary antibody was first used at 1:50 dilution (Jackson Immuno Reasearch Labs, Inc, PA, USA). Then, the cells were treated with a streptavidin AlexaFluor^®^ 488 conjugate antibody at 1:500 dilution (Thermo Fisher Scientific, Villebon sur Yvette, France).

### Image processing and analysis

For fluorescence image acquisition, confocal microscope LSM710 and ZEN software (Carl Zeiss MicroImaging, LLC, Oberkochen, Germany) were used. Images were analysed with Image J Software (nih.gov/ij). Pearson’s correlation coeficient was analyzed using “JACoP” plugin“.

### Patch clamp experiments

For electrophysiological analysis, cells were cultured in 35 mm Petri dishes at a density of 1×10^5^ cells 3 days before patch-clamp experiments, on the day 2, they have been serum-starved as predicted by the protocol. Several minutes before recording, cells were washed with the saline solution that was used for the patch-clamp experiment. Whole-cell currents were recorded as previously described (Ouadid-Ahidouch et al., 2001). The whole-cell mode of the patch-clamp technique was used with 3–5 MΩ resistance borosilicate fire-polished pipettes (Hirschmann^®^, Laborgerate, Eberstadt, Germany). Seal resistance was typically in the 3–5 GΩ range before the break of the membrane to access the whole cell configuration. Cells were voltage-clamped at −40 mV and whole cell currents were recorded following ramp-shaped membrane depolarization from −100 to +80 mV for 500 msec every 20 sec to record K^+^ currents. To record Ca^2+^ currents the cells were voltage-clamped at -40 mV and whole cell currents were recorded following ramp-shaped membrane depolarization from −100 to +100 mV for 350 msec every 2 sec. The cell under investigation was continuously superfused with control or test solutions. All electrophysiological experiments were performed at room temperature (20–22°C). External and internal solutions were of the following compositions for K^+^ currents recordings (in mM): external: NaCl 140, KCl 5, CaCl_2_ 2, MgCl_2_ 2, glucose 5 and HEPES 10 at pH 7.4 (NaOH); internal: KCl 150, MgCl_2_ 2, HEPES 10, EGTA 0.1, at pH 7.2 (KOH). For store-independent calcium currents recordings, the solutions were the following (in mM): external: Na-gluconate 142, CsCl 10, MgSO4 1.2, CaCl_2_ 2, glucose 10 and HEPES 10 at pH 7.4 (NaOH); internal: Cs-methanesulfonate 115, EGTA 10, CaCl_2_ 5, MgCl_2_ 8, HEPES 10, at pH 7.2 (CsOH).

### Manganese quenching method

To estimate divalent cation influx through the plasma membrane, we used the Manganese quenching technique as previously described [36]. MCF-7 cells were seeded on glass coverslips in 35 mm petri-dishes at a density of 10×10^4^ cells after transfection with siKv10.1, siOrai1, siKv10.1+siOrai1(siComb), siDDR1, siβ1-integrin and siControl. Mn^2+^ quenching experiments were performed 72 h post-transfection and 48 h post-starvation. Cells were loaded with 3 μM Fura-2AM in the extracellular solution supplemented with 2 mM Ca^2+^ at 37°C for 45 min. Cells were excited to 360 nm with a monochromator (TILL^®^ Photonics, Munich, Germany) and the emission signal was recorded at 510 nm by a CCD camera coupled to a Zeiss inverted microscope (Carl Zeiss MicroImaging, LLC, Oberkochen, Germany). After a period of 1 min, the Ca^2+^ (2 mM) present in the perfusion solution was replaced by 2 mM Mn^2+^. The Mn^2+^ influx was estimated from the quenching rate of 360 nm fluorescence. The extracellular solution used contained (in mM): 145 NaCl, 5 KCl, 2 CaCl_2_, 0 MgCl_2_, 10 HEPES, and 5 glucose (pH adjusted to 7.4 with NaOH).

### Apoptosis analysis

To estimate the percentage of apoptotic cells, we studied cell surface phosphatidylserine exposure, an early marker of apoptotic cell death, by performing a PE Annexin V Apoptosis Detection Kit I staining (BD Biosciences Pharmingen, Le Pont de Claix, France). Both detached and adherent cells were collected, washed twice in ice-cold PBS and re-suspended in 1× binding buffer (BD Biosciences Pharmingen, CA, USA). Apoptotic cells were determined using FITC Annexin V Apoptosis Detection Kit II (BD Biosciences Pharmingen, CA, USA). Annexin V and propidium iodide (PI) were added to the cell preparations and incubated for 15 min at 25°C in the dark. Binding buffer was then added to each tube and the samples were analyzed by flow cytometer (Accuri^®^). The following controls are used to set up compensation and quadrants: unstained cells, cells stained with FITC Annexin V (no PI) cells stained with PI (no FITC Annexin V).

### Statistical analysis

Data are presented as mean ± SEM (standard error of mean), n refers to the number of cells, and N refers to the number of cell line passages. All the experiments were performed in triplicate in at least 3 different cell lines passage number. The mean values of two groups were compared by the Student’s *t*-test or Mann-Whitney rank sum test, using Sigma-Stat 3.0 (Systat Software, Inc.), differences between the values were considered significant when *p* <0.05. The *p*-values <0.05, <0.01, and <0.001 are represented as ^*^, ^**^, and ^***^, respectively. Mean values of more than two groups were tested using two-way analysis of variance (ANOVA) followed by Holm-Sidak *post hoc* tests, differences between the values were considered significant when *p* <0.05. The *p*-values <0.05 are represented as ^*^.

## SUPPLEMENTARY MATERIALS FIGURES


