# Supplementary material for: Collagen type 1 promotes survival of human breast cancer cells by overexpressing Kv10.1 potassium and Orai1 calcium channels through DDR1-dependent pathway
Source: Oncotarget. 2017 Jul 8;9(37):24653–71. doi: 10.18632/oncotarget.19065 (PMC5973854; doi:10.18632/oncotarget.19065)
Supplement: Supplementary file 1 [file oncotarget-09-24653-s001.pdf]

Collagen type 1 promotes survival of human breast cancer cells by overexpressing Kv10.1 potassium and Orai1 calcium channels through DDR1-dependent pathway

SUPPLEMENTARY MATERIALS

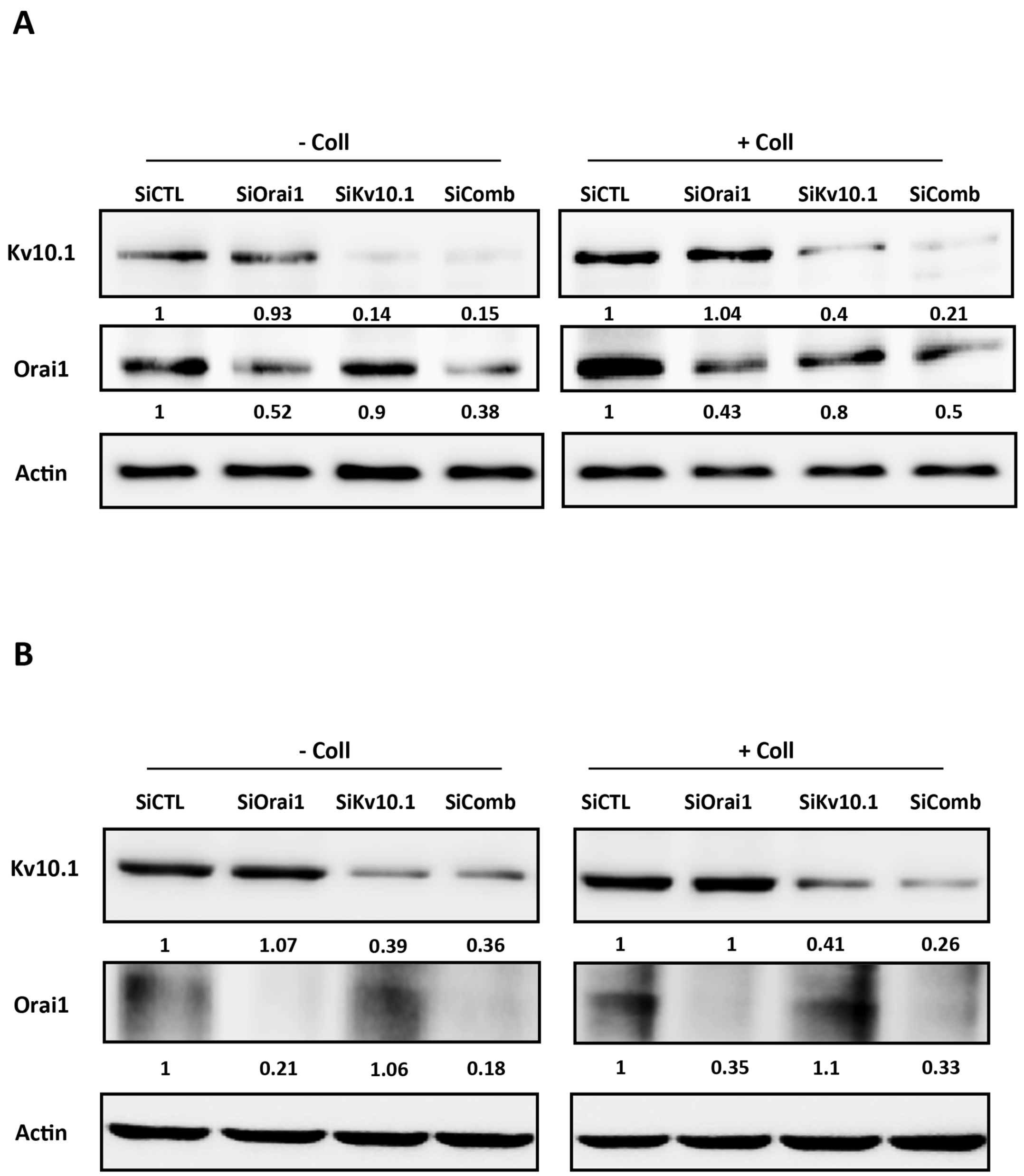

Supplementary Figure 1: Efficiency of the transfection (siKv10.1, siOrai1 and siComb (siKv10.1 + siOrai1) in MCF-7 **(A)** and T-47 D **(B)** cells. The expression of Kv10.1 and Orai1, with (+ Coll) or without collagen 1 (- Coll), were analyzed 72 h post-transfection by western blotting. Results were normalized as a percentage of the control condition (siCTL).

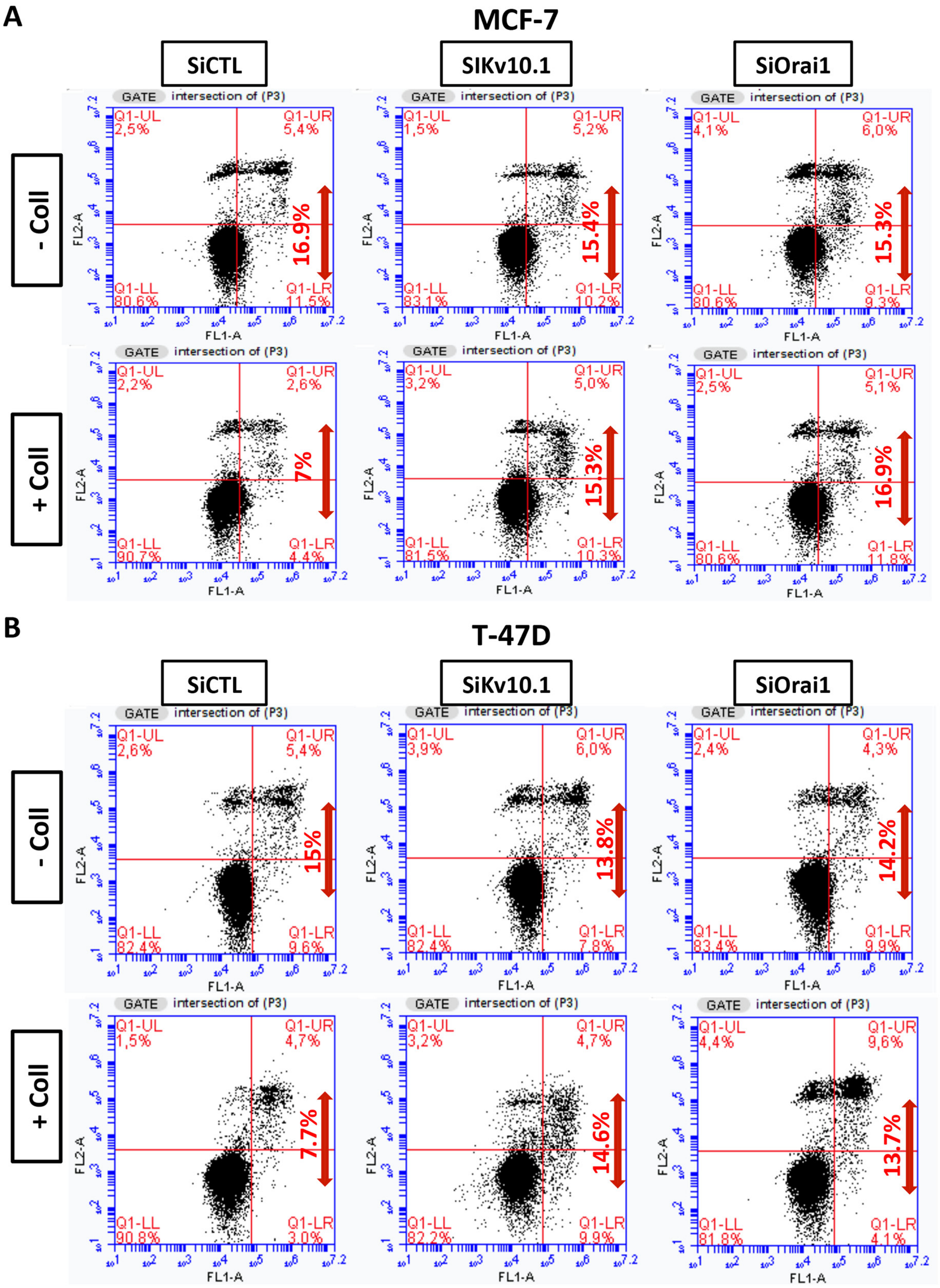

Supplementary Figure 2: Measurement of rate of apoptosis in plastic and collagen 1 conditions 48 h post-starvation by Annexin V staining in MCF-7 **(A)** and T-47D **(B)** cells transfected with siKv10.1 or siOrai1.

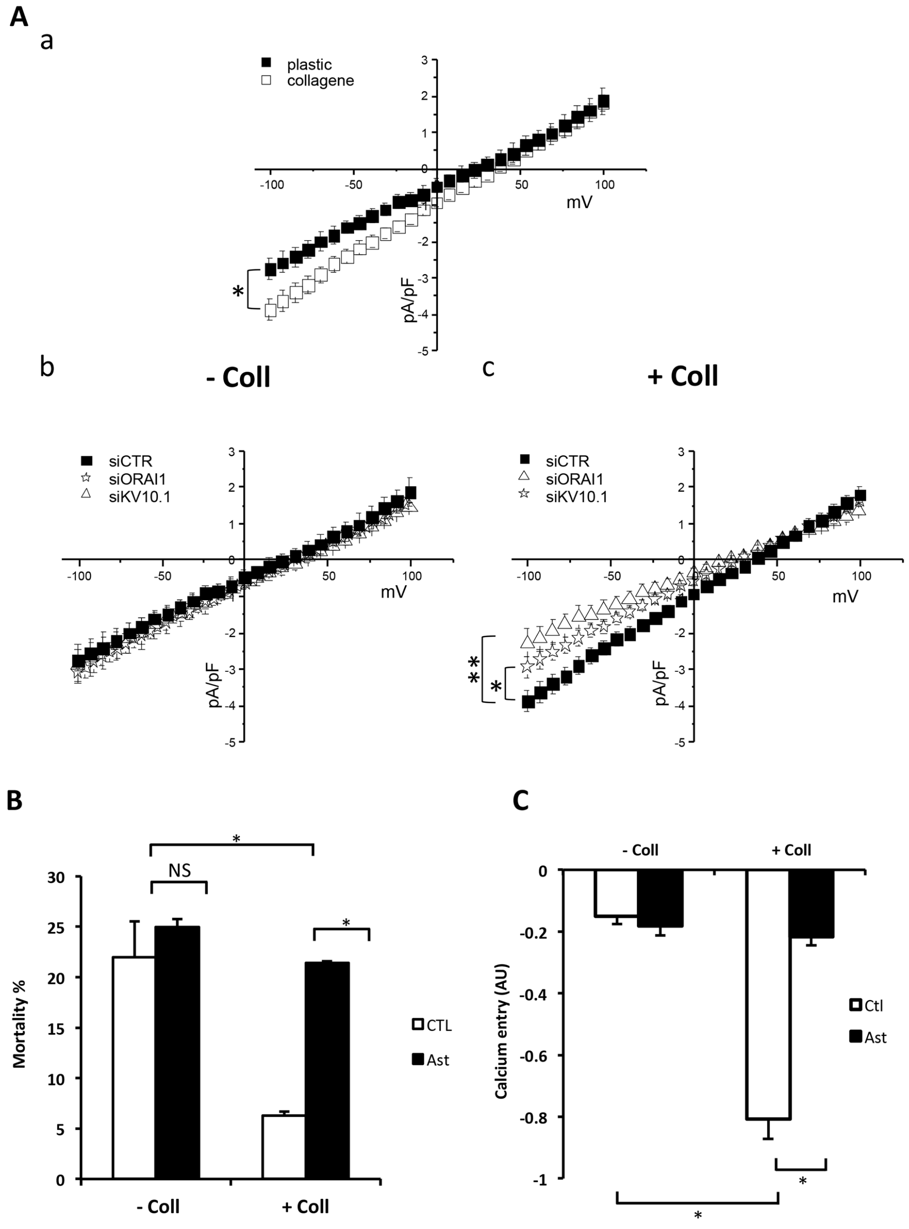

Supplementary Figure 3: Involvement of Kv10.1 and Orai1 in calcium current induced by collagen 1. (A) Collagen 1 increased Ca2+ inward current amplitude measured at -100mV **(a)**. **(b-c)** The Ca^{2+} inward current amplitude decreased only in MCF-7 cells transfected with siKv10.1 or Orai1 and coated on collagen 1. **(B)** Effect of pharmacological inhibition of Kv10.1 using astemizole (Ast) on MCF-7 cell mortality. Cells were incubated with Ast (5 µM) for 24 h and the cell mortality was measured 48 h post-starvation. Values are reported as mean {plus minus} SEM of triplicate experiments, $^{*}p<0.05$, ANOVA. - Coll: without collagen1, + Coll: with collagen 1. **(C)** Effect of pharmacological inhibition of Kv10.1 (astemizole 5 µM, Ast, for 24 h) on collagen-dependant Ca^{2+} entry by using Mn^{2+} quenching experiments. Mean slope values are reported as mean ± SEM of triplicate experiments, $^{*}p<0.05$, ANOVA. - Coll: without collagen1, + Coll: with collagen 1

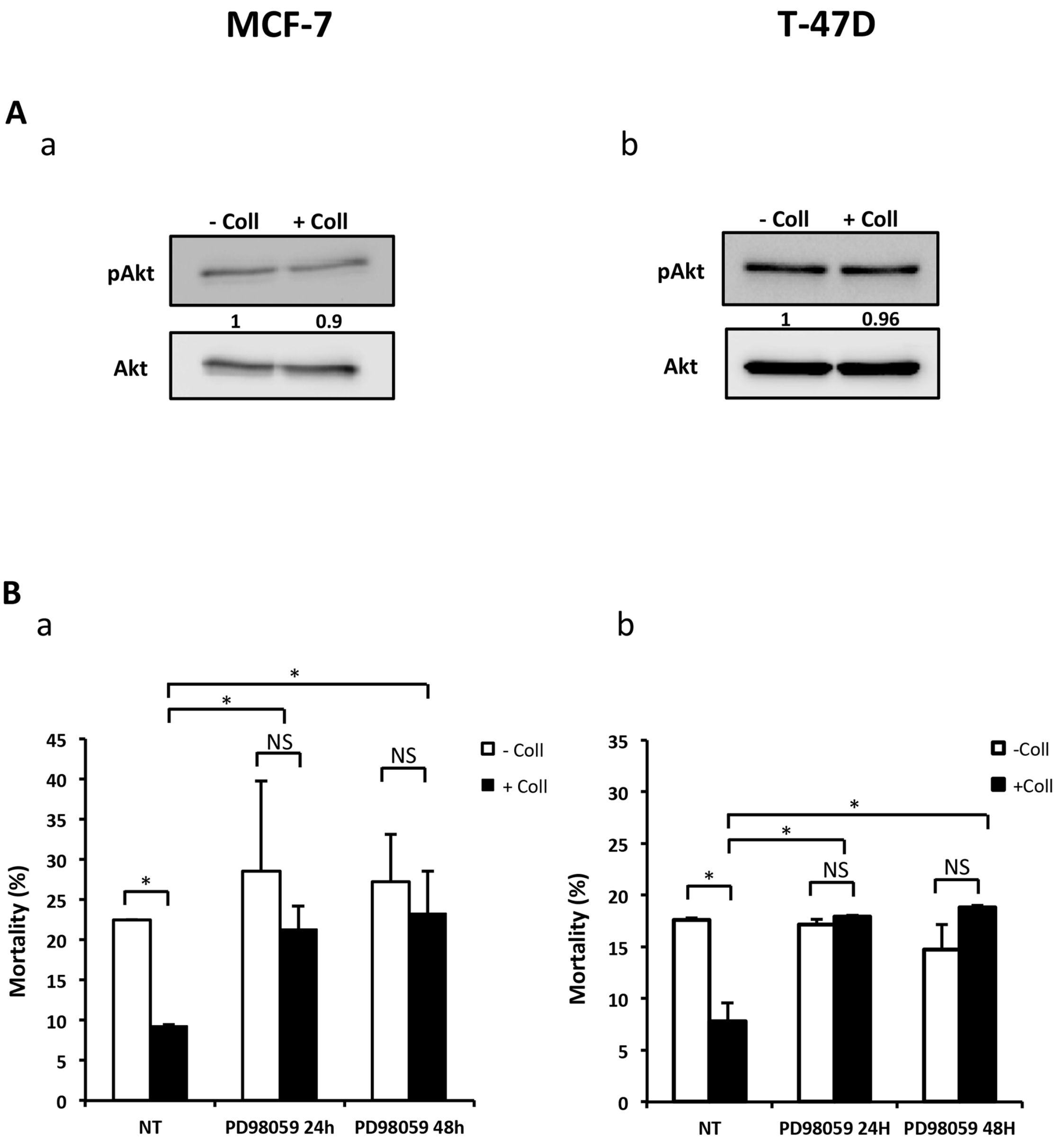

Supplementary Figure 4: (A) Collagen 1 was without effect on Akt phosphorylation in both MCF-7 **(a)** and T-47D cells **(b)**. **(B)** Effect of a 24 h or 48 h treatment by PD98059 (40 µM) on MCF-7 **(a)** and T-47 D **(b)** cells mortality. Values are reported as mean ± SEM of triplicate experiments, *p<0.05, ANOVA.

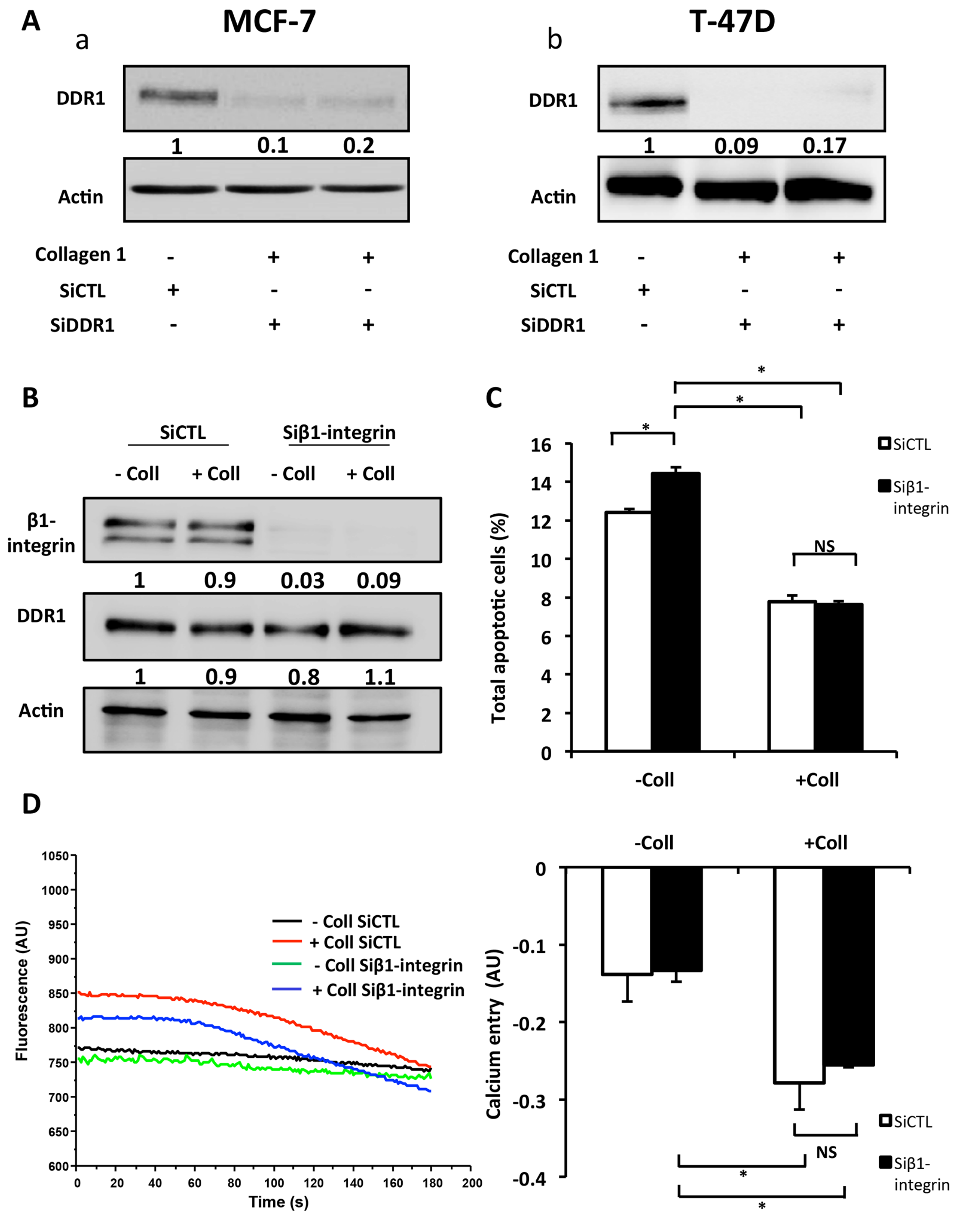

Supplementary Figure 5: (A) Representative Western Blot showing DDR1 expression in MCF-7 **(a)** and T-47D **(b)** cells 48 h post-starvation and the transfection efficiency of siDDR1 72 h post-transfection. **(B-D)** Transfection efficiency of β1-integrin, and the effect on DDR1 expression (B), apoptosis rate (C) and calcium entry (D) in MCF-7 cells seeded or not on collagen 1.

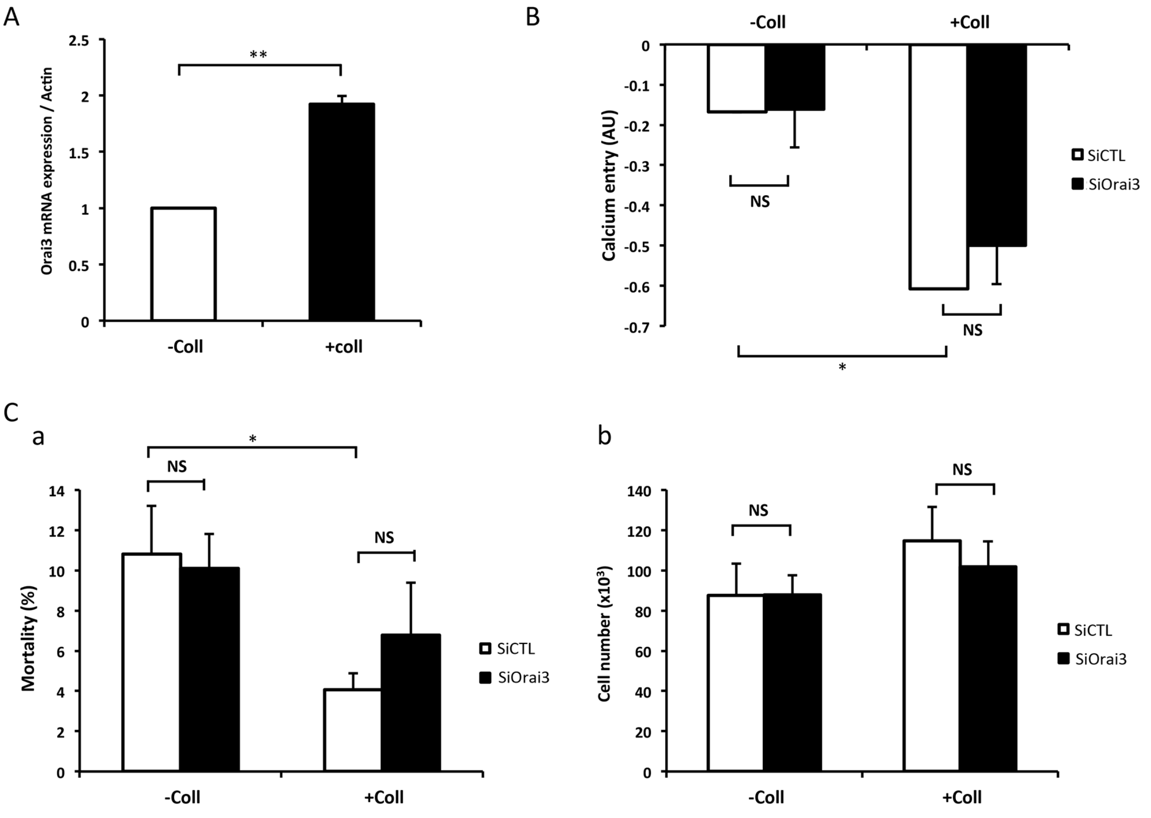

Supplementary Figure 6: (A) Effect of Collagen 1 on Orai3 expression. **(B)** Effect of Orai3 silencing on basal Ca^{2+} influx by using Mn^{2+} quenching experiments. Mean slope values are reported as mean ± SEM of triplicate experiments, $^{*}p$<0.05, ANOVA. **(C)** Effect of Orai3 silencing on cell mortality **(a)** and viability **(b)**. Cells were starved for 48 h. The mortality and the viability were measured by Trypan Blue, values are reported as mean ± SEM of triplicate experiments, $^{*}p$<0.05, ANOVA.

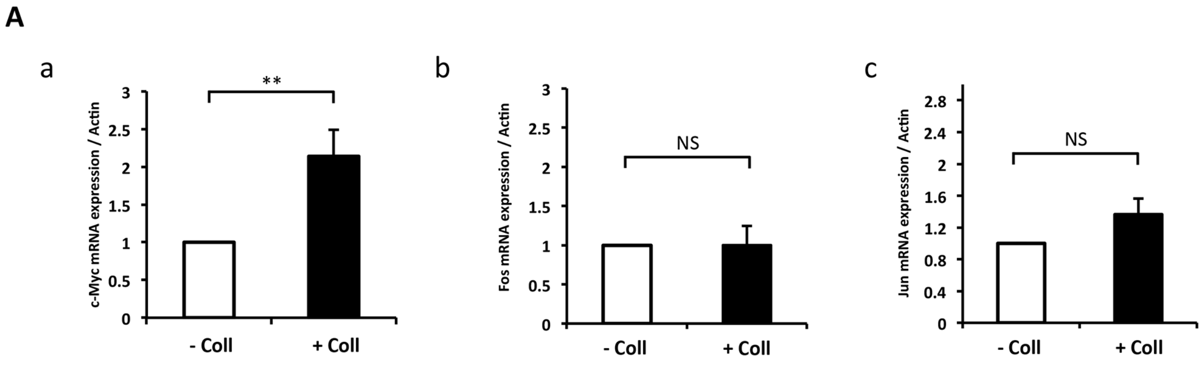

Supplementary Figure 7: (A) Expression of c-Myc **(a)**, Fos **(c)** and Jun **(d)** mRNA by using qPCR in MCF-7 cells 48 h post-starvation in the presence (+ Coll) or not (- Coll) of collagen 1. Results were normalized as a percentage of the control condition (without collagen) and reported as mean ± SEM of triplicate experiments, $^{**}p$<0.01, Student's *t*-test.
